# Percutaneous intervention versus coronary artery bypass graft surgery in left main coronary artery stenosis: a systematic review and meta-analysis

**DOI:** 10.1186/s12916-017-0853-1

**Published:** 2017-04-21

**Authors:** Xin-Lin Zhang, Qing-Qing Zhu, Jing-Jing Yang, Yu-Han Chen, Yang Li, Su-Hui Zhu, Jun Xie, Lian Wang, Li-Na Kang, Biao Xu

**Affiliations:** 1 Department of Cardiology, Affiliated Drum Tower Hospital, Nanjing University School of Medicine, 321 Zhongshan Road, 210008 Nanjing, Jiangsu Province China; 2 Department of Respiratory Medicine, Jinling Hospital, Nanjing University School of Medicine, Nanjing, China; 3 Department of Traditional Chinese Medicine, Nanjing Drum Tower Hospital, Clinical College of Traditional Chinese and Western Medicine, Nanjing University of Chinese Medicine, Nanjing, China

**Keywords:** Left main coronary artery disease, Percutaneous coronary intervention, Coronary artery bypass graft surgery, Meta-analysis, Randomized controlled trials, Matched observational studies

## Abstract

**Background:**

The optimal revascularization technique in patients with left main coronary artery disease (CAD) remains controversial. We aimed to compare the long-term performance of percutaneous coronary intervention (PCI) versus coronary artery bypass graft (CABG) surgery in treatment of left main CAD.

**Methods:**

PubMed, EMBASE, and the Cochrane Library were searched until November 16, 2016.

**Results:**

Six randomized controlled trials and 22 matched observational studies including 22,487 patients and 90,167 patient-years of follow-up were included. PCI was associated with an overall higher risk for the major adverse cardiac and cerebrovascular events (hazard ratio (HR), 1.42; 95% confidence interval (CI), 1.14–1.77), mainly driven by higher rates of myocardial infarction (HR, 1.69; 95% CI, 1.22–2.34) and revascularization (HR, 2.80; 95% CI, 1.86–4.22). The overall risks for all-cause death (HR, 1.05; 95% CI, 0.93–1.20), cardiac death (HR, 1.05; 95% CI, 0.69–1.59), stroke (HR, 0.64; 95% CI, 0.33–1.24), and the composite safety endpoint of death, myocardial infarction, or stroke (HR, 1.06; 95% CI, 0.97–1.16) were similar between PCI and CABG. Stratified analysis based on stent types showed that the increased risk for myocardial infarction associated with PCI was only evident in patients with bare-metal stents or early-generation drug-eluting stents (DES), but not newer-generation DES. Stratified analyses based on study designs showed largely similar findings with the overall analyses, except for a significantly higher incidence of myocardial infarction in adjusted studies (HR, 2.01; 95% CI, 1.64–2.45) but a trend toward higher incidence in randomized trials (HR, 1.39; 95% CI, 0.85–2.27) associated with PCI.

**Conclusions:**

Compared with CABG, PCI with newer-generation DES might be a safe alternative revascularization strategy for treatment of left main CAD, but is associated with more repeat revascularization.

**Electronic supplementary material:**

The online version of this article (doi:10.1186/s12916-017-0853-1) contains supplementary material, which is available to authorized users.

## Background

Left main coronary artery disease (CAD) is associated with poor clinical outcomes; without revascularization, the 3-year mortality reaches 50% [1]. Coronary artery bypass graft (CABG) surgery has been the standard treatment for patients with left main CAD for a long time [2]. However, the past few decades have witnessed rapid advances in percutaneous coronary intervention (PCI), including stent technology, adjunctive imaging support, and pharmacotherapy [3], which substantially changed the revascularization strategy for treating left main CAD. The most recent European and US guidelines made a recommendation with a Class II to I indication for PCI in left main CAD patients with low to intermediate anatomic complexity [4, 5].

However, these guidelines were mainly based on midterm findings of the SYNTAX trial [6], the PRECOMBAT trial [7], and two other small trials [8, 9], all of which were underpowered to determine the comparative safety and efficacy of PCI versus CABG, particularly for individual hard endpoints. In the last 3 years, long-term follow-up data of the PRECOMBAT and SYNTAX trials [10, 11], the large-scale EXCEL (Evaluation of Xience Everolimus-Eluting Stent Versus Coronary Artery Bypass Surgery for Effectiveness of Left Main Revascularization; NCT01205776), the NOBLE (Coronary Artery Bypass Grafting vs Drug Eluting Stent Percutaneous Coronary Angioplasty in the Treatment of Unprotected Left Main Stenosis; NCT01496651) trials [12, 13], and several large-scale adjusted registries [14–16] were published. Notably, the EXCEL and NOBLE trials may be the last clinical trials randomizing patients with left main CAD to PCI or CABG. In this context, we performed a systematic review and meta-analysis of randomized controlled trials (RCTs) and matched observational studies to compare the long-term performance of PCI versus CABG in patients with left main CAD, and to determine whether the development of stents would affect these findings.

## Methods

This meta-analysis was conducted in accordance with the Preferred Reporting Items for Systematic Reviews and Meta-Analyses (PRISMA) guideline (Additional file 1) [17].

### Data sources and searches

We searched PubMed, the Cochrane Central Register of Controlled Trials, and EMBASE from their inception to November 16, 2016, without language restrictions. The following keywords were used: “left main” and “percutaneous coronary intervention” and “coronary artery bypass”. The comprehensive search strategy is provided in Additional file 2. Reference lists of the identified reports and relevant reviews were manually screened by one reviewer (XZ) to identify further relevant studies.

### Study selection

Two reviewers (XZ and JY) independently screened the titles and abstracts for eligibility, and retrieved the full-text of citations with potential relevance. Any discrepancies were resolved by a third investigator (YC). All studies included in the meta-analysis had to be RCTs or matched observational studies making direct comparisons of PCI with CABG in patients with left main coronary artery stenosis. Additionally, studies had to contain at least 100 patients and report outcomes of interest with an at least 12 months’ follow-up to minimize the small study effects, due to the rarity of cardiovascular events. Observational studies had to provide adjusted estimates by either propensity matching or propensity score adjustment or multivariate adjustment to minimize the bias from baseline confounding factors. We excluded duplicated publications, animal studies, review articles, studies not comparing PCI with CABG, studies conducted in mixed population in which data for left main CAD could not be abstracted, studies with small sample size and short follow-up duration, and studies reporting unadjusted results.

### Outcome measures

Primary clinical endpoints were all-cause mortality and cardiovascular mortality; secondary end points were myocardial infarction, stroke, repeat revascularization, a composite of major adverse cardiac and cerebrovascular events (MACCE; all-cause death, myocardial infarction, stroke, or repeat revascularization), and a composite safety endpoint of all-cause death, myocardial infarction, or stroke. Definitions of outcomes from each randomized trial were provided (Additional file 2: Table S1).

### Data extraction and quality assessment

All data were extracted by two investigators (XZ and JY) from each study, which included the study author, publication date, number of patients, duration of study follow-up, study enrolling period, adjustment method, patient age and sex, baseline EUROSCORE and SYNTAX score, baseline ejection fraction, the percentage of patients with multi-vessel disease, diabetes, hypertension, and the risk for each study outcome, etc. Three reviewers (XZ, QZ, and JY) independently evaluated the potential risk of bias of randomized trials by applying the Cochrane Collaboration’s tool [18] and the quality of observational studies by using the Newcastle–Ottawa Scale criteria [19].

### Data synthesis and statistical analysis

Hazard ratios (HR) and their corresponding 95% confidence intervals (CIs) were directly retrieved from each trial and matched study. We used HR as the statistic estimate because it correctly reflects the nature of the data and accounts for censoring. The extent of heterogeneity was assessed with the Cochran Q test and the *I* ^*2*^ statistic, with *I* ^*2*^ values of 25%, 50%, and 75% indicating low, moderate, and high heterogeneity, respectively [20]. If significant heterogeneity was found across studies (*P* < 0.10 or *I* ^*2*^ > 50%), we calculated pooled risks by using a random-effects model and the DerSimonian–Laird method [21]; otherwise, a fixed-effects model with the Mantel–Haenszel method was used [22]. Stratified analyses were performed for trials and matched studies, for newer-generation drug-eluting stents (DES) and bare-metal stents (BMS) or early-generation DES, and for midterm (1–3 years) and long-term (>3 years) follow-up. A test for subgroup differences was performed across the examined subgroups with meta-regression analysis. For a subgroup analysis of trials, we also extracted the raw data for each outcome of interest to calculate the odds ratio (OR). Potential publication bias was examined by performing Begg’s and Egger’s tests. All statistical analyses were performed with the Stata software, version 12.0 (STATA Corporation, College Station, TX, USA).

## Results

### Study selection and characteristics

Additional file 2: Figure S1 shows the flow diagram of the study selection. We identified 2597 citations though database searches; 28 studies reported in 26 articles met inclusion criteria. Six studies were randomized trials [10–13, 23, 24] and 22 were matched observational studies. Seven studies were carried out in propensity matching populations [16, 25–30], 9 studies provided adjusted estimates by propensity score adjustment [14, 31–38], and 6 studies made multivariate adjustment [15, 39–41]. Long-term outcome data were available in all trials (range, 3–10 years; mean, 5.5 years) and 14 matched studies (range, 3–9.7 years; mean, 4.6 years). The risks of bias for trials and matched studies were generally low to moderate, as presented in Additional file 2: Table S2 and Table S3. The nature of the intervention made trials blinded for clinicians or patients impossible, but this was not considered a source of significant bias.

A total of 22,487 patients (90,167 patient-years of follow-up) receiving PCI (n = 10,406) or CABG (n = 12,081) were included in the analysis. Patients enrolled were mainly men (77.2%), with a median age of 65.3 years (range, 61–82 years). Overall, roughly one third of patients presented with diabetes, half had hyperlipidemia, two thirds had hypertension, and one third were active smokers. When reported, the mean value of left ventricular ejection fraction ranged from 40% to 65%. The median EUROSCORE was 4.3 (range, 1.8–8.4) for PCI group and 5.0 (range, 2.0–9.5) for the CABG group. Detailed baseline characteristics of each study were presented in Table 1 and Additional file 2: Table S4. Main inclusion and exclusion criteria, primary and secondary endpoints of randomized trials were presented in Additional file 2: Table S5.

**Table 1:** Selected baseline characteristics of comparative randomized controlled trials and matched observational studies

Trial	Year	Age, years	Male, %	Diabetes, %	Hypertension, %	Dyslipidemia, %	Current smoker, %	Prior PCI, %	Prior MI, %	No.	FU, years	EF, %	Study period	Adjusting method
LE MANS [24]	2016	61.3 ± 8.4	73	17	70	60	NA	NA	33	105	10	53.7 ± 6.7	2001–2004	Randomization
PRECOMBAT [10]	2015	62.7 ± 9.5	77	30	51.3	40	27.7	12.7	6.7	600	5	60.8 ± 8.5	2004–2009	Randomization
SYNTAX [11]	2014	65.6 ± 10.1	75.6	25.6	62.4	75.4	24	NA	25.4	705	5	NA	2005–2007	Randomization
Boudriot et al. [23]	2011	69 (63–73)	77	33	82	64	28	NA	14	201	5	65 (55–68)	2003–2009	Randomization
EXCEL [12]	2016	65.9 ± 9.5	77.5	28	73.9	69.3	20.8	15.9	16.9	1905	3	57.3 ± 9.0	2010–2014	Randomization
NOBLE [13]	2016	66.2 ± 9.4	76	15	66	78	22	20	NA	1184	5	60 (52–64)	2008–2015	Randomization
Zheng et al. [14]	2016	62.2 ± 9.1	82	31	64.3	59.1	53.6	9.7	38.1	4046	3	60.2 ± 8.2	2004–2010	Propensity score adjustment
Yu et al. [33]	2016	64 (57–70)	82.5	28.7	58.9	34.6	44.9	6.8	21.7	922	7.1	62 (54–68)	2003–2009	Propensity score adjustment
Lu et al. [32]	2016	69 ± 11	85.5	46	83	50	67	NA	NA	478	4.3	49 ± 12	2004–2010	Propensity score adjustment
Wei et al. [41]	2016	71.0 ± 5.9	79	45.2	72.6	21.0	58.1	NA	NA	126	1.3	48.8 ± 7.5	2012–2013	Multivariate adjusted
IRIS-MAIN registry-1 [15]	2016	61 ± 10	75.3	31.5	51.8	33.4	33.1	10.8	15.5	954	9.7	58 ± 11	1995–2002	Multivariate adjusted
IRIS-MAIN registry-2 [15]	2016	64 ± 9	74.4	38.4	54.8	34.6	27.2	12.3	13.3	1901	5.6	56 ± 11	2003–2006	Multivariate adjusted
IRIS-MAIN registry-3 [15]	2016	65 ± 9	79.1	42.5	65.9	50.3	25.9	13.4	12.2	2362	3	55 ± 11	2007–2013	Multivariate adjusted
Jeong et al. [28]	2013	60.8 ± 10	79.2	30.8	49.1	1.7	20.1	18.2	6.9	318	4.7	NA	2001–2009	Propensity matching
DELTA registry [16]	2012	66.8 ± 10.5	65	31.6	68.1	65.6	43	16.8	NA	1204	3.5	53.2 ± 11.4	2002–2006	Propensity matching
CREDO-Kyoto 2 [40]	2012	69.4 ± 9.2	77	45	85	NA	25	NA	16	1005	3	60.2 ± 13.4	2005–2007	Multivariate adjusted
Chang et al. [27]	2012	64 ± 8.7	72.6	35.8	53.2	33.2	26.3	14.7	12.6	380	4.2	40.1 ± 27.8	2003–2009	Propensity matching
Yi et al. [29]	2012	64.2 ± 8.3	82.7	31.3	62.5	NA	NA	NA	NA	256	5	NA	2003–2007	Propensity matching
CUSTOMIZE registry [25]	2011	65.3 ± 10.5	76.3	33.5	68.2	54.3	48	22	31.2	346	3	52.1 ± 9.0	2002–2011	Propensity matching
Rittger et al. [34]	2011	65 ± 7	78	45	84	94	NA	22	18	286	1	55 ± 14	2004–2007	Propensity score adjustment
Asan-Multivessel Registry [31]	2011	NA	NA	NA	NA	NA	NA	NA	NA	550	5.6	NA	2003–2005	Propensity score adjustment
Kang et al. [30]	2010	64.3 ± 10.3	71.4	41.9	67.6	52.4	48.6	NA	NA	210	2.8	53.7 ± 13.1	2003–2006	Propensity matching
Mäkikallio et al. [39]	2009	70 ± 9	80	17	46	NA	18	3	NA	287	1	54 ± 11	2005–2007	Multivariate adjusted
MAIN-COMPARE registry [26]	2008	64 (56–70)	71.2	33	50	30.1	27.1	15.1	10	1084	3	61 (55–66)	2000–2006	Propensity matching
Ghenim et al. [35]	2009	79.6 ± 3.5	71.7	23.6	65.1	41.5	24.5	20.7	19.8	211	1	NA	2004–2006	Propensity score adjustment
White et al. [36]	2008	69.4 ± 1.7	77	27	76	77	17	NA	NA	353	2	54 ± 11	2003–2007	Propensity score adjustment
Rodes-Cabau et al. [37]	2008	82 ± 2	63	26	72	82	6	8	59	249	2	56 ± 14	2002–2008	Propensity score adjustment
Palmerini et al. [38]	2007	78 (75–88)	66	26	73	47	39	NA	NA	259	2	53 (25–78)	2003–2006	Propensity score adjustment

Values are mean, median (interquartile range) or %. *EF* ejection fraction, *FU* follow-up, *MI* myocardial infarction, *NA* not available, *PCI* percutaneous coronary intervention

Expanded study abbreviations are as follows: CREDO-Kyoto 2 = the Coronary Revascularization Demonstrating Outcome Study in Kyoto (CREDO-Kyoto) PCI/CABG Registry Cohort-2; CUSTOMIZE = the Appraise a Customized Strategy for Left Main Revascularization Registry; DELTA = the drug-eluting stent for left main coronary artery disease registry; EXCEL = the Evaluation of XIENCE versus Coronary Artery Bypass Surgery for Effectiveness of Left Main Revascularization trial; IRIS-MAIN = Interventional Research Incorporation Society-Left MAIN Revascularization registry; LE MANS = Study of UnprotectedLeft Main Stenting Versus Bypass Surgery; MAIN-COMPARE = Revascularization for Unprotected Left Main Coronary Artery Stenosis: Comparison of Percutaneous Coronary Angioplasty versus Surgical Revascularization registry; NOBLE = The Nordic-Baltic-British left main revascularisation study; PRECOMBAT = the Premier of Randomized Comparison of Bypass Surgery versus Angioplasty Using Sirolimus-Eluting Stent in Patients with Left Main Coronary Artery Disease trial; SYNTAX = the other Synergy between Percutaneous Coronary Intervention with Taxus and Cardiac Surgery trial

### Total and cardiovascular mortality

**Fig. 1:**
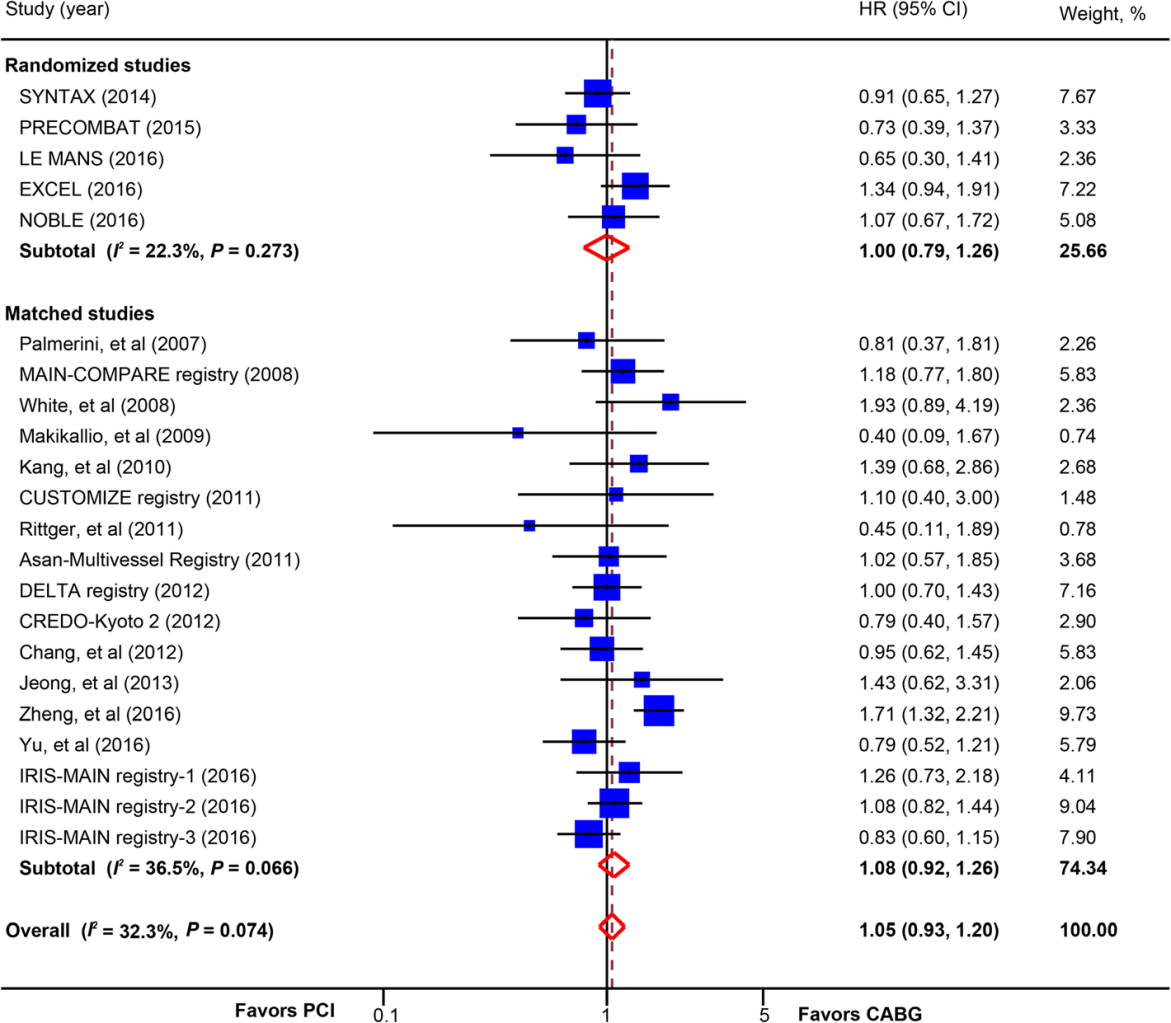
Pooled risk for all-cause mortality with percutaneous coronary intervention (PCI) versus coronary artery bypass graft (CABG) by study design. *CI* confidence interval, *HR* hazard ratio. CREDO-Kyoto 2 = the Coronary Revascularization Demonstrating Outcome Study in Kyoto (CREDO-Kyoto) PCI/CABG Registry Cohort-2; CUSTOMIZE = the Appraise a Customized Strategy for Left Main Revascularization Registry; DELTA = the drug-eluting stent for left main coronary artery disease registry; EXCEL = the Evaluation of XIENCE versus Coronary Artery Bypass Surgery for Effectiveness of Left Main Revascularization trial; IRIS-MAIN = Interventional Research Incorporation Society-Left MAIN Revascularization registry; LE MANS = Study of Unprotected Left Main Stenting Versus Bypass Surgery; MAIN-COMPARE = Revascularization for Unprotected Left Main Coronary Artery Stenosis: Comparison of Percutaneous Coronary Angioplasty versus Surgical Revascularization registry; NOBLE = the Nordic-Baltic-British left main revascularisation study; PRECOMBAT = the Premier of Randomized Comparison of Bypass Surgery versus Angioplasty Using Sirolimus-Eluting Stent in Patients with Left Main Coronary Artery Disease trial; SYNTAX = the other Synergy between Percutaneous Coronary Intervention with Taxus and Cardiac Surgery trial

There was no significant difference in all-cause mortality between PCI and CABG (22 studies, 20,966 patients; HR, 1.05; 95% CI, 0.93–1.20) (Fig. 1). The lack of statistically significant difference was consistent across multiple stratified analyses (Table 2): in the subgroup of trials (5 trials, 4499 patients; HR, 1.00; 95% CI, 0.79–1.26) and matched studies (17 studies, 16,467 patients; HR, 1.08; 95% CI, 0.92–1.26); in studies with newer-generation DES (3 studies, 5451 patients; HR, 1.05; 95% CI, 0.78–1.42) and those with BMS or early-generation DES (19 studies, 15515 patients; HR, 1.05; 95% CI, 0.91–1.22); and in studies with long-term follow-up (17 studies, 19571 patients; HR, 1.05; 95% CI, 0.92–1.20) and midterm follow-up (5 studies, 1395 patients; HR, 1.02; 95% CI, 0.60–1.74). No significant interaction was detected in these stratified analyses. Subgroup analysis with raw data from trials to calculate OR also did not find significant difference in total mortality between PCI and CABG (6 trials, 4700 patients; OR, 1.02; 95% CI, 0.83–1.26) (Additional file 2: Figure S2).

**Table 2:** Stratified analysis of each endpoint based on study design, duration of follow-up, and stent type

Endpoint	Subgroup	No. of study	HR	95% LCI	95% UCI	*P* value	*I* ^*2*^	*P*_heterogeneity	*P*_interaction
All-cause death	Randomized trial	5	1.00	0.79	1.26	0.97	22.3	0.27	0.57
	Matched study	17	1.08	0.92	1.26	0.36	36.5	0.07	
	Long-term	17	1.05	0.92	1.20	0.44	35	0.08	0.93
	Midterm	5	1.02	0.6	1.74	0.95	37.1	0.17	
	Second-generation DES	3	1.05	0.78	1.42	0.75	47.7	0.15	0.99
	BMS or early-generation DES	19	1.05	0.91	1.22	0.50	33.3	0.08	
Cardiac death	Randomized trial	4	1.00	0.72	1.39	0.99	20.5	0.29	0.62
	Matched study	5	1.08	0.51	2.29	0.83	77.9	0.001	
	Long term	8	1.09	0.72	1.65	0.67	73.1	< 0.001	0.31
	Mid term	1	0.28	0.03	2.70	0.27	NA	NA	
	Second-generation DES	2	1.10	0.75	1.63	0.63	0	0.59	0.97
	BMS or early-generation DES	7	1.01	0.58	1.77	0.97	77.2	< 0.001	
MI	Randomized trial	5	1.39	0.85	2.27	0.19	57.5	0.05	0.18
	Matched study	5	2.01	1.64	2.45	< 0.001	0	0.72	
	Long term	10	1.69	1.22	2.24	0.002	57.7	0.01	NA
	Mid term	0	NA	NA	NA	NA	NA	NA	
	Second-generation DES	2	1.56	0.52	4.71	0.43	87.3	0.005	0.39
	BMS or early-generation DES	8	1.92	1.59	2.31	< 0.001	0	0.73	
Stroke	Randomized trial	5	0.84	0.47	1.50	0.56	50.9	0.09	0.25
	Matched study	4	0.44	0.18	1.07	0.07	79.4	0.002	
	Long term	9	0.64	0.33	1.24	0.19	83.5	< 0.001	NA
	Mid term	0	NA	NA	NA	NA	NA	NA	
	Second-generation DES	2	1.25	0.44	3.54	0.68	74.6	0.05	0.15
	BMS or early-generation DES	7	0.48	0.25	0.93	0.03	74.2	0.001	
Revascularization	Randomized trial	5	1.68	1.40	2.02	< 0.001	0	0.53	0.10
	Matched study	15	3.52	2.07	5.99	< 0.001	93.8	< 0.001	
	Long term	18	2.57	1.68	4.92	< 0.001	93.5	< 0.001	0.17
	Mid term	2	8.69	3.33	22.69	< 0.001	0	0.93	
	Second-generation DES	3	2.26	1.23	4.15	0.008	86.2	0.001	0.72
	BMS or early-generation DES	17	2.92	1.80	4.75	< 0.001	93.4	< 0.001	
Death/MI/stroke	Randomized trial	3	0.96	0.80	1.15	0.66	0	0.86	0.43
	Matched study	14	1.10	0.99	1.22	0.08	39.5	0.06	
	Long term	14	1.07	0.97	1.17	0.16	36.4	0.09	0.48
	Mid term	3	0.89	0.58	1.38	0.61	11.9	0.32	
	Second-generation DES	2	0.96	0.80	1.15	0.69	0	0.62	0.52
	BMS or early-generation DES	15	1.10	0.99	1.22	0.09	35.3	0.09	
MACCE	Randomized trial	5	1.20	1.00	1.44	0.05	44.6	0.13	0.31
	Matched study	11	1.57	1.14	2.17	0.006	87.7	< 0.001	
	Long term	12	1.50	1.18	1.91	0.001	86.7	< 0.001	0.39
	Mid term	4	1.07	0.52	2.19	0.86	80.2	0.002	
	Second-generation DES	3	1.35	1.15	1.59	< 0.001	28.6	0.25	0.93
	BMS or early-generation DES	13	1.42	1.05	1.92	0.02	87.3	< 0.001	

*BMS* bare-metal stent, *DES* drug-eluting stent, *HR* hazard ratio, *LCI* lower 95% confidence interval, *MACCE* major adverse cardiac and cerebrovascular event, *MI* myocardial infarction, *NA* not applicable, *UCI* upper 95% confidence interval

**Fig. 2:**
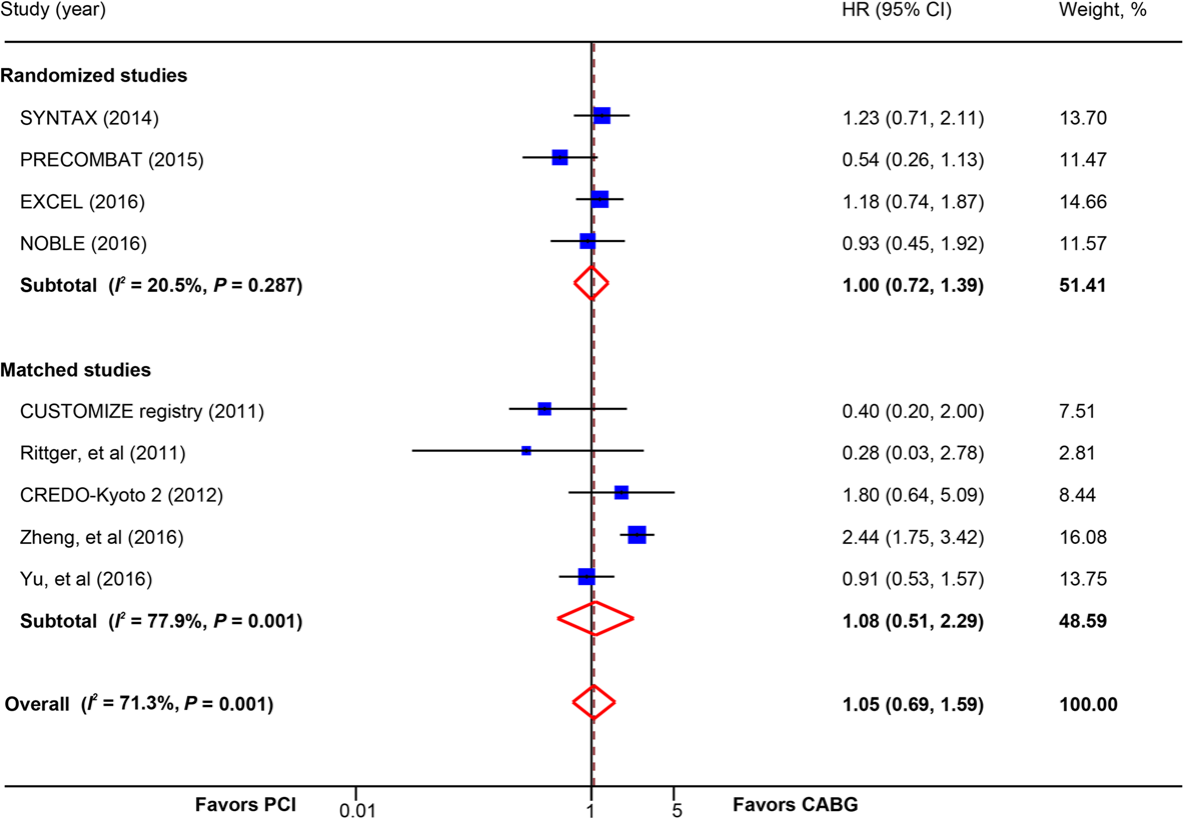
Pooled risk for cardiovascular mortality with percutaneous coronary intervention (PCI) versus coronary artery bypass graft (CABG) by study design. *CI* confidence interval; *HR* hazard ratio. CREDO-Kyoto 2 = the Coronary Revascularization Demonstrating Outcome Study in Kyoto (CREDO-Kyoto) PCI/CABG Registry Cohort-2; CUSTOMIZE = the Appraise a Customized Strategy for Left Main Revascularization Registry; EXCEL = the Evaluation of XIENCE versus Coronary Artery Bypass Surgery for Effectiveness of Left Main Revascularization trial; NOBLE = the Nordic-Baltic-British left main revascularisation study; PRECOMBAT = the Premier of Randomized Comparison of Bypass Surgery versus Angioplasty Using Sirolimus-Eluting Stent in Patients with Left Main Coronary Artery Disease trial; SYNTAX = the other Synergy between Percutaneous Coronary Intervention with Taxus and Cardiac Surgery trial

Cardiovascular mortality did not differ between PCI and CABG (9 studies, 10,999 patients; HR, 1.05; 95% CI, 0.69–1.59) (Fig. 2). Consistent findings were observed in the subgroup of trials (HR, 1.00; 95% CI, 0.72–1.39) and matched studies (HR, 1.08; 95% CI, 0.51–2.29) and other subgroups (Table 2). Subgroup analysis in trials calculating OR also did not find significant difference in cardiovascular mortality between PCI and CABG (OR, 1.03; 95% CI, 0.77–1.38) (Additional file 2: Figure S3).

### Myocardial infarction, revascularization, and stroke

**Fig. 3:**
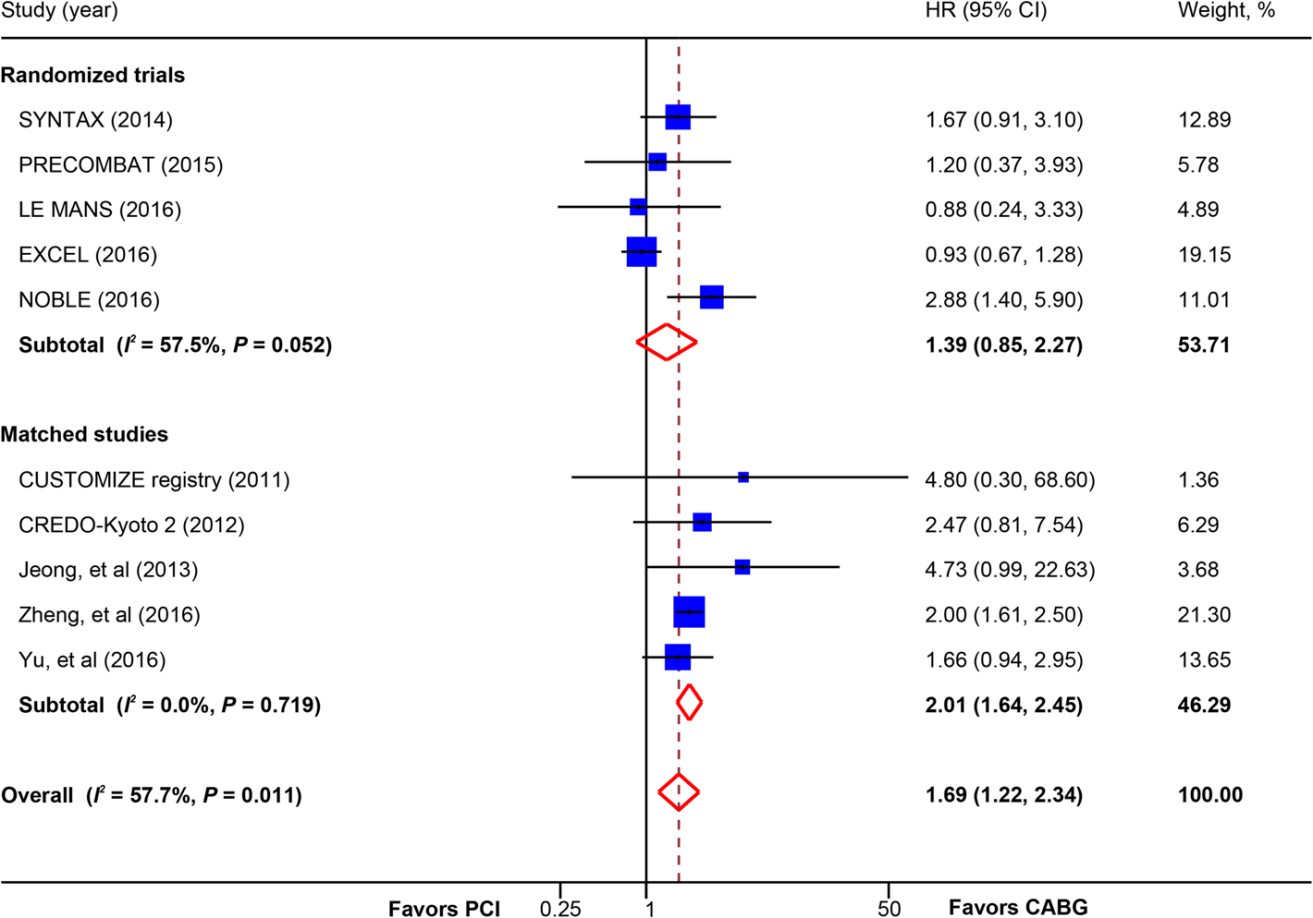
Pooled risk for myocardial infarction with percutaneous coronary intervention (PCI) versus coronary artery bypass graft (CABG) by study design. *CI* confidence interval, *HR* hazard ratio. CREDO-Kyoto 2 = the Coronary Revascularization Demonstrating Outcome Study in Kyoto (CREDO-Kyoto) PCI/CABG Registry Cohort-2; CUSTOMIZE = the Appraise a Customized Strategy for Left Main Revascularization Registry; EXCEL = the Evaluation of XIENCE versus Coronary Artery Bypass Surgery for Effectiveness of Left Main Revascularization trial; LE MANS = Study of Unprotected Left Main Stenting Versus Bypass Surgery; NOBLE = the Nordic-Baltic-British left main revascularisation study; PRECOMBAT = the Premier of Randomized Comparison of Bypass Surgery versus Angioplasty Using Sirolimus-Eluting Stent in Patients with Left Main Coronary Artery Disease trial; SYNTAX = the other Synergy between Percutaneous Coronary Intervention with Taxus and Cardiac Surgery trial

Overall, there was a statistically significant increased risk of myocardial infarction in patients receiving PCI compared with CABG (10 studies, 11,136 patients; HR, 1.69; 95% CI, 1.22–2.34) (Fig. 3). In analysis stratified by study design, a trend toward increased risk was found in trials (5 trials, 4499 patients; HR, 1.39; 95% CI, 0.85–2.27) whereas a statistically significant increase was found in matched studies (5 studies, 6637 patients; HR, 2.01; 95% CI, 1.64–2.45). Further analysis revealed that this difference was mainly driven by a higher rate of myocardial infarction in patients receiving BMS or early-generation DES (HR, 1.92; 95% CI, 1.59–2.31), but not those receiving newer-generation DES (HR, 1.56; 95% CI, 0.52–4.71) (Table 2). Subgroup analysis in trials calculating OR showed a trend toward increased risk of myocardial infarction in PCI group (6 trials, 4700 patients; OR, 1.44; 95% CI, 0.90–2.30) (Additional file 2: Figure S4).

**Fig. 4:**
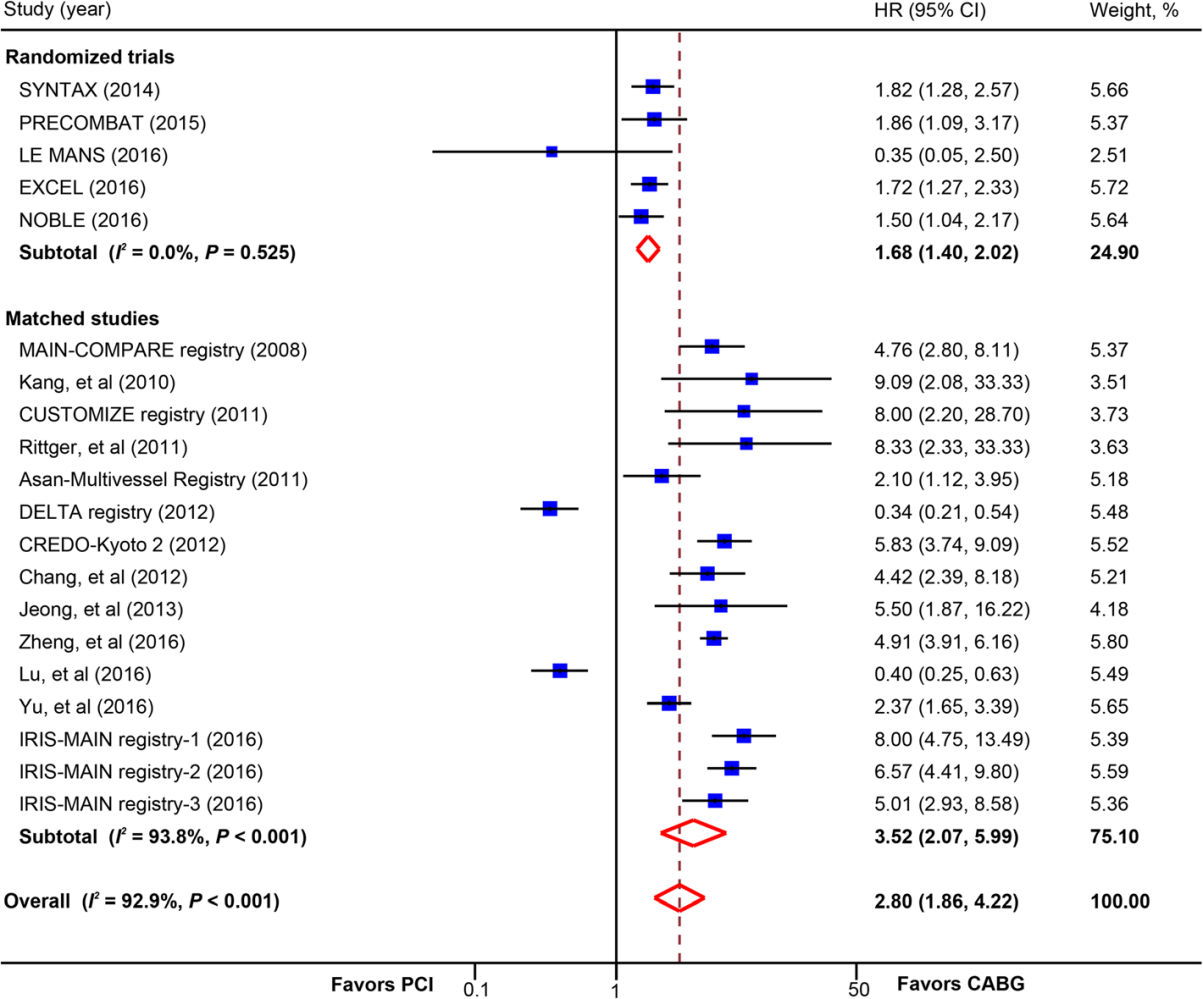
Pooled risk for revascularization with percutaneous coronary intervention (PCI) versus coronary artery bypass graft (CABG) by study design. *CI* confidence interval; *HR* hazard ratio. CREDO-Kyoto 2 = the Coronary Revascularization Demonstrating Outcome Study in Kyoto (CREDO-Kyoto) PCI/CABG Registry Cohort-2; CUSTOMIZE = the Appraise a Customized Strategy for Left Main Revascularization Registry; DELTA = the drug-eluting stent for left main coronary artery disease registry; EXCEL = the Evaluation of XIENCE versus Coronary Artery Bypass Surgery for Effectiveness of Left Main Revascularization trial; IRIS-MAIN = Interventional Research Incorporation Society-Left MAIN Revascularization registry; LE MANS = Study of Unprotected Left Main Stenting Versus Bypass Surgery; MAIN-COMPARE = Revascularization for Unprotected Left Main Coronary Artery Stenosis: Comparison of Percutaneous Coronary Angioplasty versus Surgical Revascularization registry; NOBLE = the Nordic-Baltic-British left main revascularisation study; PRECOMBAT = the Premier of Randomized Comparison of Bypass Surgery versus Angioplasty Using Sirolimus-Eluting Stent in Patients with Left Main Coronary Artery Disease trial; SYNTAX = the other Synergy between Percutaneous Coronary Intervention with Taxus and Cardiac Surgery trial

There was a statistically significant increased risk of revascularization in the PCI group compared with CABG (20 studies, 20,545 patients; HR, 2.80; 95% CI, 1.86–4.22) (Fig. 4). This finding was consistent in trials (5 trials, 4499 patients; HR, 1.68; 95% CI, 1.40–2.02) and matched studies (15 studies, 16,046 patients; HR, 3.52; 95% CI, 2.07–5.99), in patients receiving BMS or early-generation DES and newer-generation DES, and in studies with long-term follow-up and mid-term follow-up (Table 2). No significant interaction was detected between these subgroups. Subgroup analysis in trials calculating OR also showed similar findings (6 trials, 4700 patients; OR, 1.78; 95% CI, 1.48–2.14) (Additional file 2: Figure S5).

Overall, there was no statistically significant difference in stroke between PCI and CABG (9 studies, 10,790 patients; HR, 0.64; 95% CI, 0.33–1.24) (Additional file 2: Figure S6). However, in matched observational studies, there was a trend toward lower stroke rate in favor of PCI (HR, 0.44; 95% CI, 0.18–1.07; *P* = 0.07) and in patients receiving BMS or early-generation DES, this benefit was significant (HR, 0.48; 95% CI, 0.25–0.93) (Table 2). Subgroup analysis in trials calculating OR showed no significant difference in incidence of stroke (6 trials, 4700 patients; OR, 0.85; 95% CI, 0.57–1.26) (Additional file 2: Figure S7).

### Composite outcomes

**Fig. 5:**
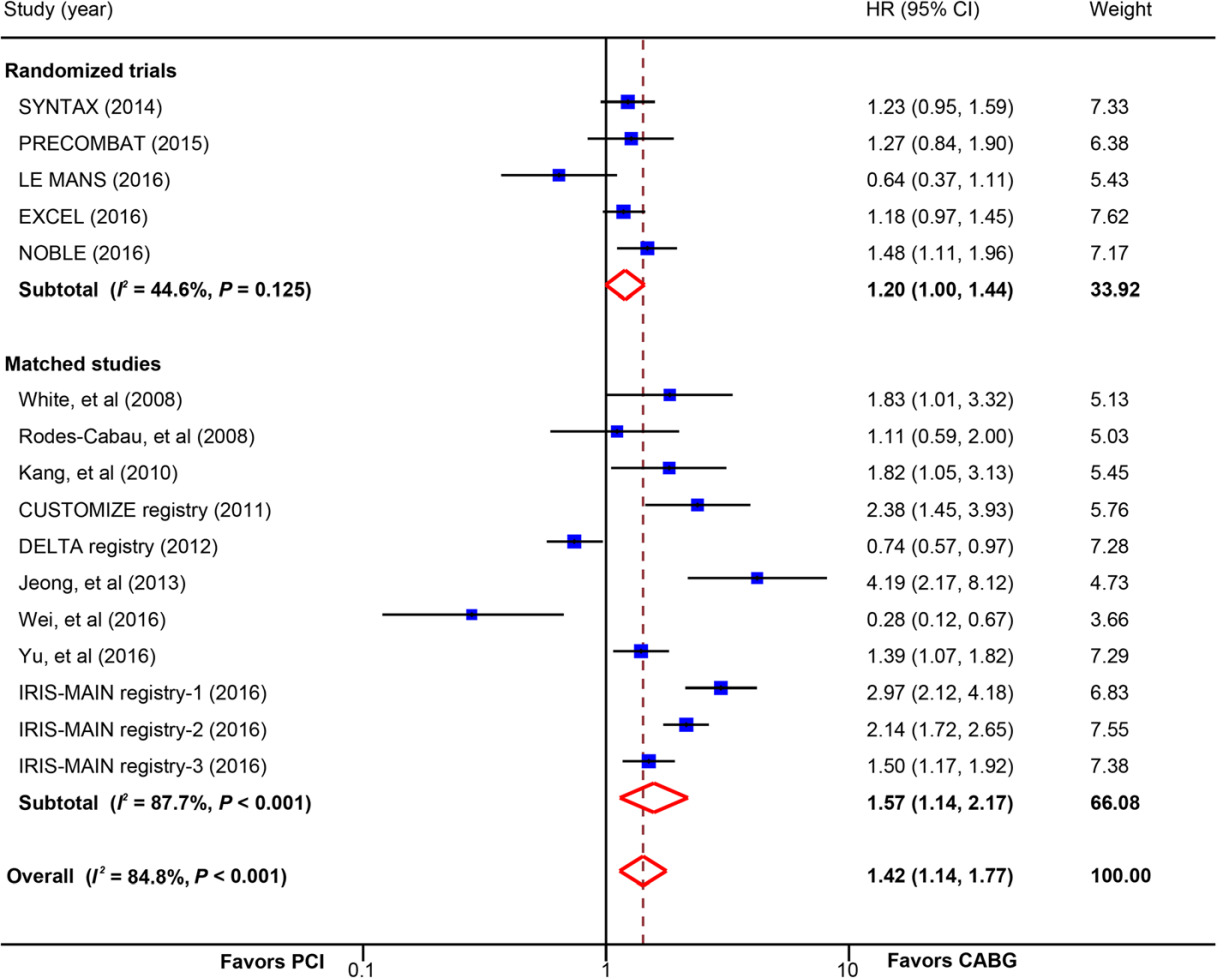
Pooled risk for major adverse cardiac and cerebrovascular events (MACCE) with percutaneous coronary intervention (PCI) versus coronary artery bypass graft (CABG) by study design. *CI* confidence interval, *HR* hazard ratio. CUSTOMIZE = the Appraise a Customized Strategy for Left Main Revascularization Registry; DELTA = the drug-eluting stent for left main coronary artery disease registry; EXCEL = the Evaluation of XIENCE versus Coronary Artery Bypass Surgery for Effectiveness of Left Main Revascularization trial; IRIS-MAIN = Interventional Research Incorporation Society-Left MAIN Revascularization registry; LE MANS = Study of Unprotected Left Main Stenting Versus Bypass Surgery; NOBLE = the Nordic-Baltic-British left main revascularisation study; PRECOMBAT = the Premier of Randomized Comparison of Bypass Surgery versus Angioplasty Using Sirolimus-Eluting Stent in Patients with Left Main Coronary Artery Disease trial; SYNTAX = the other Synergy between Percutaneous Coronary Intervention with Taxus and Cardiac Surgery trial

Overall, there was a statistically significant increased risk of MACCE in patients receiving PCI compared with CABG (16 studies, 13,444 patients; HR, 1.42; 95% CI, 1.14–1.77) (Fig. 5). In the analysis stratified by study design, a statistically significant increase was found both in trials (5 trials, 4499 patients; HR, 1.20; 95% CI, 1.00–1.44) and in matched studies (11 studies, 8945 patients; HR, 1.57; 95% CI, 1.14–2.17); in studies with newer-generation DES (3 studies, 5451 patients; HR, 1.35; 95% CI, 1.15–1.59) and those with BMS or early-generation DES (13 studies, 7993 patients; HR, 1.42; 95% CI, 1.05–1.92). No significant interaction was detected between these subgroups (Table 2). Subgroup analysis in trials with raw data to calculate OR also showed a statistically significant increased risk in PCI group (6 trials, 4700 patients; OR, 1.34; 95% CI, 1.16–1.54) (Additional file 2: Figure S8).

**Fig. 6:**
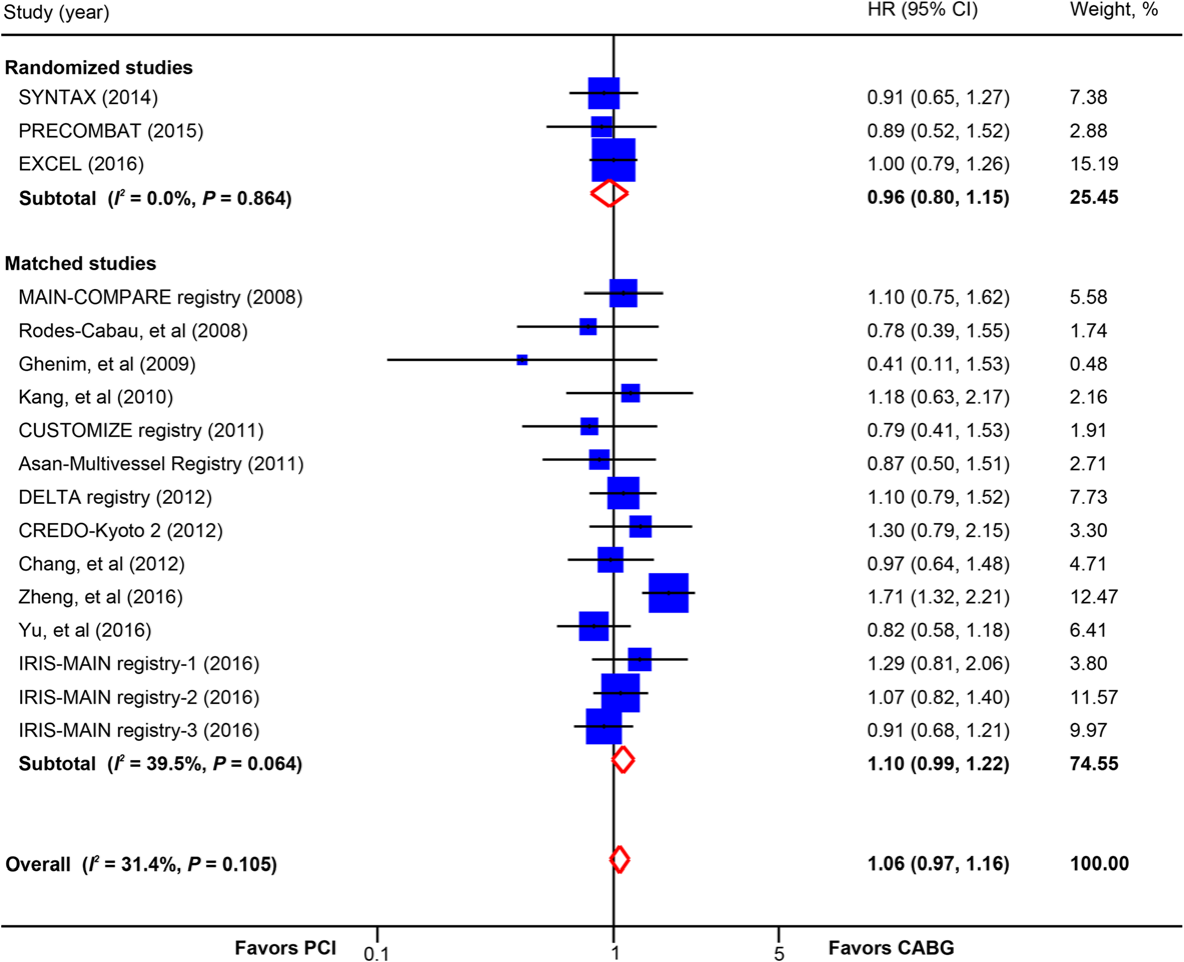
Pooled risk for the composite endpoint of death, myocardial infarction, and stroke with percutaneous coronary intervention (PCI) versus coronary artery bypass graft (CABG) by study design. *CI* confidence interval, *HR* hazard ratio. CREDO-Kyoto 2 = the Coronary Revascularization Demonstrating Outcome Study in Kyoto (CREDO-Kyoto) PCI/CABG Registry Cohort-2; CUSTOMIZE = the Appraise a Customized Strategy for Left Main Revascularization Registry; DELTA = the drug-eluting stent for left main coronary artery disease registry; EXCEL = the Evaluation of XIENCE versus Coronary Artery Bypass Surgery for Effectiveness of Left Main Revascularization trial; IRIS-MAIN = Interventional Research Incorporation Society-Left MAIN Revascularization registry; MAIN-COMPARE = Revascularization for Unprotected Left Main Coronary Artery Stenosis: Comparison of Percutaneous Coronary Angioplasty versus Surgical Revascularization registry; PRECOMBAT = the Premier of Randomized Comparison of Bypass Surgery versus Angioplasty Using Sirolimus-Eluting Stent in Patients with Left Main Coronary Artery Disease trial; SYNTAX = the other Synergy between Percutaneous Coronary Intervention with Taxus and Cardiac Surgery trial

There was no statistically significant difference in the composite safety endpoint of death, myocardial infarction, or stroke between PCI and CABG (17 studies, 18,634 patients; HR, 1.06; 95% CI, 0.97–1.16) (Fig. 6). In patients receiving BMS or early-generation DES, there was a trend toward higher rate of this composite endpoint in favor of CABG (HR, 1.10; 95% CI, 0.99–1.22; *P* = 0.09) (Table 2). Subgroup analysis in trials calculating OR showed no significant difference in rate of this composite safety endpoint (3 trials, 3210 patients; OR, 0.98, 95% CI, 0.81–1.20) (Additional file 2: Figure S9).

## Discussion

On the basis of pooled data from 28 studies that included nearly 22,500 patients (over 90,000 patient-years of follow-up) with left main CAD receiving PCI or CABG treatment, we found that PCI was associated with a higher risk for MACCE, which was evident both in studies with newer-generation DES and those with BMS or early-generation DES, mainly driven by higher rates of myocardial infarction and revascularization associated with PCI. The overall risks for all-cause death, cardiac death, stroke, and the composite safety endpoint of death, myocardial infarction, or stroke were similar between PCI and CABG. Stratified analysis showed that the increased risk for myocardial infarction associated with PCI was only evident in patients with BMS or early-generation DES but not newer-generation DES.

Our study has several strengths compared with other reviews [42–44]. First, we included available randomized trials and matched observational studies in the literature to improve the power and reliability of our results. Our report remains the largest database on treatment choice of left main CAD ever analyzed. Such comprehensive literature search made stratified analyses based on important factors possible. Other reviews did not include many recently published large-scale RCTs or registries, enrolled only small-to-moderate number of patients, and were unable to perform important sensitivity analysis based on stent types, etc. [42–45]. Second, we made restricted inclusion and exclusion criteria to decrease the risk of bias. We excluded many available observational studies in unmatched populations or without statistical adjustment to minimize bias from confounding factors. We only included studies with over 100 patients and reporting outcomes with at least 1-year follow-up to minimize the small study effects due to the rarity of cardiovascular events. Several other reviews included unadjusted studies, combining randomized or adjusted studies with these [43, 44]. Potential bias could not be avoided in their analyses. Additionally, the meta-analysis of Alam et al. [44] did not set inclusion criteria on the number of patients and duration of follow-up; in their analysis, observational studies with 20 patients in one arm or 6-month follow-up was included. Third, we selected HR as the statistic estimate because it incorporates censoring and time frame, and thus reflects on the nature of survival data. We also performed subgroup analyses of randomized trials calculating OR from raw events to validate the results. The majority of other meta-analyses did not take into account the variation of follow-up across studies, which was actually very large [44, 46]. Absence of adjustment for this variation could cause potential bias. The study of Athappan et al. [43] did perform sensitivity analysis by pooling hazard ratios, but their analysis was confined to less than five studies, mostly including only two to three studies in one analysis, making results of sensitivity analysis inaccurate. Fourth, we concluded data from long-term follow-up, made stratified analysis according to the generation of stent, and detected the different performance of newer-generation DES and BMS or early-generation DES as compared with CABG. Again, no other meta-analyses performed or reported the difference between different generations of stents [42–45]. Fifth, analyses of data from long-term follow-up findings on all outcomes were largely consistent across all subgroups. The lack of interaction in subgroups internally confirmed the robustness of our findings.

Our analysis and other reviews [42–45] showed consistent finding that PCI was not associated with an increase in total mortality compared with CABG in treating left main CAD. The conclusion was reinforced by the fact that our findings were consistent across subgroup analyses based on different study design, different generation of stents, and different duration of follow-up, which was not performed in other reviews [42–45]. We consider this finding important because total mortality is the most important safety endpoint of clinical trials. This observation provided the most basic safety support for the use of PCI in left main CAD because it does not increase mortality.

Concerning the broader safety of PCI versus CABG in treating left main CAD, our and a few other meta-analyses [44] evaluated the composite safety endpoint of all-cause death, myocardial infarction, or stroke. Our study suggested no difference between PCI with newer-generation DES and CABG, but a trend toward disadvantage in PCI with BMS or early-generation DES, when compared with CABG. By contrast, the study of Alam et al. [44] showed significantly lower rate of this safety endpoint in favor of PCI (OR, 0.63; 95% CI, 0.49–0.82). However, their work must be interpreted with critical caution because of their nature of bias from confounding factors (combined analyses of adjusted and a body of unadjusted studies) and because they did not incorporate the remarkable variation of follow-up within studies. Meanwhile, they enrolled only one third of the patients, most of whom were from unadjusted observational studies (5722 patients versus 18,634 patients in our study). Without causing serious safety outcomes, PCI with newer-generation DES might be a safe alternative revascularization strategy for treatment of left main CAD, especially for those patients who refuse bypass surgery due to the fear of thoracotomy and wound healing. However, it should also be noted that, in the EXCEL trial [12], the largest RCT on this topic, more safety events occurred in the PCI group between 30 days and 3 years than the CABG group (11.5% versus 7.9%, *P* = 0.02). Therefore, the broad safety of PCI with newer-generation DES versus CABG still needs to be confirmed from longer-term follow-up data.

Our overall analysis demonstrated a statically significant difference in rate of myocardial infarction in favor of CABG in long-term follow-up. A similar finding was also observed in the large NOBLE trial [13], which, however, adopted a different definition of myocardial infarction from others studies by excluding periprocedural myocardial infarction in their analysis. We performed a sensitivity analysis by excluding the NOBLE trial, and found that PCI was still associated with an increased risk for myocardial infarction (HR, 1.58; 95% CI, 1.12–2.23). This observation, however, was not consistent in several prior meta-analyses, probably due to their insufficient power [43–45] or large potential bias from confounding factors [44]. For instance, in the study of Athappan et al. [43], myocardial infarction showed a statistically significant trend in favor of CABG in analysis including unadjusted data, but this trend disappeared when the analysis was confined to adjusted data; however, this only contained one to three studies and less than 1500 patients in one analysis. It was notable in our study that PCI-associated high risk for myocardial infarction was only evident in patients receiving BMS or early-generation DES, but not those with newer-generation DES. This is accordant with the concept that rate of myocardial infarction decreases following stent technology development [47]. Therefore, if PCI is to be performed in patients with left main CAD, a newer-generation DES should be preferred.

A number of meta-analysis showed an overall decreased stroke risk in the PCI arm compared with CABG in patients with left main CAD [43–45]. However, our overall analysis suggested a similar incidence between PCI and CABG, even though a benefit in favor of PCI was seen in patients receiving BMS or early-generation DES. Similar to our findings, in depth analysis of the NOBLE trial [13] and the overall SYNTAX trial (included left main and three-vessel CAD) [48] challenged the true risk benefit of PCI by showing that PCI was associated with an increase in late stroke, which might completely counteract the early benefit of PCI [13]. The reasons why a benefit was observed in BMS and early-generation DES but not newer-generation DES still remain unclear.

Our analysis showed a consistent finding with other reviews and randomized trials [12, 13] or registry data [14, 15, 26] that PCI was associated with a remarkably increased risk for revascularization compared with CABG. Although the rapid development of stent technology from BMS to early-generation DES and then to newer-generation DES clearly decreased the rate of revascularization [47], our subgroup analysis demonstrated persistently higher risk for revascularization in the PCI arm irrespective of the stent types. The comparative risk still needs to be investigated in longer-term follow-up, when graft fail becomes obvious in the CABG arm [49].

Our study demonstrated a remarkable benefit in favor of CABG with respect to MACCE risk in treating left main CAD. This finding is consistent in subgroups with the BMS or early-generation DES and newer-generation DES, and supported by the NOBLE trial [13] and a patient-level meta-analysis of the PRECOMBAT and SYNTAX trials [45]. The increased incidence for MACCE in the PCI arm was mainly driven by higher risk for myocardial infarction and revascularization, but no obvious risk advantage in stroke associated with PCI. Such directional consistency of the individual component outcomes in our study improved the reliability of our analysis and made interpretation of the MACCE endpoint clear.

### Limitations

We acknowledge several limitations. First, the results were analyzed on trial level data but not on patient level data. Second, definitions of clinical outcomes other than mortality were based on the definitions in the original studies and thus were not completely uniform across these studies. Third, the reporting of EUROSCORE and SYNTAX score were absent in a large portion of studies, making meta-regression analyses of the effects of these variables on clinical outcomes inaccurate and was therefore not performed. Fourth, selective outcome reporting was observed in a number of observational studies, and publication bias was observed in several outcome analyses. Fifth, heterogeneity is evident in the analyses of certain outcomes. We made several subgroup and meta-regression analyses to explore the heterogeneity, and used random-effects models to incorporate heterogeneity among studies.

## Conclusions

Pooled data from nearly 22,500 patients (over 90,000 patient-years of follow-up) with left main CAD receiving revascularization treatment suggest that PCI is associated with a higher risk for MACCE in long-term follow-up, mainly driven by higher rates of myocardial infarction and revascularization. The increased risk for myocardial infarction associated with PCI was only evident in patients receiving BMS or early-generation DES but not those receiving newer-generation DES. Therefore, compared with CABG, PCI with newer-generation DES might be a safe alternative revascularization strategy for treatment of left main CAD, but is associated with more repeat revascularization.

## Additional files

Additional file 1: PRISMA checklist. (DOCX 29 kb)

Additional file 2: Supplemental information. (DOCX 1488 kb)

## Abbreviations

BMS: bare-metal stent
CABG: coronary artery bypass graft surgery
CAD: coronary artery disease
CI: confidence interval
DES: drug-eluting stents
HR: hazard ratio
MACCE: major adverse cardiac and cerebrovascular events
OR: odds ratio
PCI: percutaneous coronary intervention
