# Supplementary material for: Percutaneous intervention versus coronary artery bypass graft surgery in left main coronary artery stenosis: a systematic review and meta-analysis
Source: BMC Med. 2017 Apr 21;15:84. doi: 10.1186/s12916-017-0853-1 (PMC5399381; doi:10.1186/s12916-017-0853-1)
Supplement: Supplementary file 2 — Supplemental information. (DOCX 1488 kb) [file 12916_2017_853_MOESM2_ESM.docx]

**Additional file 2**

**Percutaneous Intervention versus Coronary Artery Bypass Graft Surgery in Left Main Coronary Artery Stenosis:**

**A Systematic Review and Meta-analysis**

**Running title:** PCI versus CABG in Left Main CAD

Xin-Lin Zhang^1†^, Qing-Qing Zhu^2†^, Jing-Jing Yang^3†^, Yu-Han Chen^1^, Yang Li^1^, Su-Hui Zhu^1^, Jun Xie^1^, Lian Wang^1^, Li-Na Kang^1^ and Biao Xu^1^

^1^Department of Cardiology, Affiliated Drum Tower Hospital, Nanjing University School of Medicine, Nanjing, China; ^2^Department of Respiratory Medicine, Jinling Hospital, Nanjing University School of Medicine, Nanjing, China; ^3^Department of Traditional Chinese Medicine, Nanjing Drum Tower Hospital, Clinical College of Traditional Chinese and Western Medicine, Nanjing University of Chinese Medicine, Nanjing, China.

^†^X-LZ, Q-QZ and J-JY contributed equally to this work.

Correspondence to Biao Xu (xubiao@medmail.com.cn) and Li-Na Kang (army0001@163.com), Department of Cardiology, Affiliated Drum Tower Hospital, Nanjing University School of Medicine, 321 Zhongshan Road, 210008 Nanjing, Jiangsu Province, China. Tel: +86 25 831 052 05; fax: +86 25 833 0 80 59.

**Table of contents**

**Search Algorithm**

**Table S1.** Outcome definition in randomized controlled trials.

**Figure S1.** Flow diagram of study selection.

**Table S2.** Risk of bias of included randomized controlled trials.

**Table S3.** Study quality of included matched observational studies using the Newcastle-Ottawa scale.

**Table S4.** Other selected baseline characteristics of comparative randomized controlled trials and matched observational studies.

**Table S5.** Main inclusion and exclusion criteria, primary and secondary endpoints of randomized controlled trials.

**Table S6.** Stratified analysis of each endpoint based on duration of follow-up and study design.

**Figure S2.** Pooled odds ratio for total mortality with percutaneous coronary intervention versus coronary artery bypass graft surgery.

**Figure S3.** Pooled odds ratio for cardiovascular mortality with percutaneous coronary intervention versus coronary artery bypass graft surgery.

**Figure S4.** Pooled odds ratio for myocardial infarction with percutaneous coronary intervention versus coronary artery bypass graft surgery.

**Figure S5.** Pooled odds ratio for revascularization with percutaneous coronary intervention versus coronary artery bypass graft surgery.

**Appendix Figure S6.** Pooled hazard ratio for stroke with percutaneous coronary intervention versus coronary artery bypass graft surgery.

**Figure S7.** Pooled odds ratio for stroke with percutaneous coronary intervention versus coronary artery bypass graft surgery.

**Figure S8.** Pooled odds ratio for the composite outcome of death, stroke, myocardial infarction and revascularization with percutaneous coronary intervention versus coronary artery bypass graft surgery.

**Figure S9.** Pooled odds ratio for the composite outcome of death, stroke and myocardial infarction with percutaneous coronary intervention versus coronary artery bypass graft surgery.

**Search Algorithm**

"Left main" AND ("percutaneous coronary intervention" OR PCI OR stent*) AND ("coronary artery bypass" OR CABG OR "bypass surgery" OR "coronary bypass")

**Table S1.** Outcome definition in randomized controlled trials.

| **NOBLE (The Nordic-Baltic-British left main revascularisation study)**  **All–cause mortality:** Death from any cause.  **Cardiac death:** Cardiac death was defined as any death due to a suspected cardiac cause (myocardial infarction, low–output heart failure, fatal arrhythmia), unwitnessed death and death of unknown cause. All procedure–related deaths, including those related to concomitant treatment, were classified as cardiac death. The endpoint was included post hoc. (Modified from Cutlip et al. Circulation. 2007;115:2344–2351) The information on cause of death was obtained from hospital patient files, from general practitioners, or from families if no other source was available.  **Vascular death:** Death caused by non–coronary vascular causes, including cerebrovascular disease, pulmonary embolism, ruptured aortic aneurysm, dissecting aneurysm, or other vascular diseases. The endpoint was included post hoc. (Modified from Cutlip et al. Circulation. 2007;115:2344–2351)  Non–procedure–related myocardial infarction: A rise in biochemical markers exceeding the decision limit for myocardial infarction (99th percentile including < 10% CV) with at least one of the following; (1) ischemic symptoms, (2) ECG changes indicative of ischemia (ST segment elevation or depression), and (3) development of a pathologic Q–wave with no relation to a PCI procedure.  **Repeat revascularization:** Any new PCI or CABG operation performed during follow–up. If an index revascularisation was attempted or successful, any subsequent revascularisation was counted as repeat revascularisation. Attempted PCI was defined as an advancement of a wire in the coronary tree at least. Attempted CABG was defined as at least initiation of an index operation.  **Procedure–related biomarker release:** The diagnosis of a procedure–related biomarker increase required a rise in total creatine kinase (CK) and/or Troponin–T/I. Due to the great heterogeneity of biomarkers and various assays used during the study in participating centres, this comparison was omitted from the final analysis.  **Procedural myocardial infarction:** Diagnosis of procedural MI for both PCI and CABG patients was based on CK–MB elevations when available. Patients needed to have stable angina pectoris as the clinical indication OR a normal baseline CK–MB, TnI, TnT, or highly sensitive TnT, to be assessable for procedural MI. Diagnosis required a CK–MB value above 10 x URL or ULN to establish the diagnosis. The diagnosis could also be placed by the combination of a CK–MB value above 5 x URL or ULN, AND one or more of the following: (1) new pathological Q waves in at least 2 contiguous leads or new persistent non–rate–related left bundle branch block, or (2) angiographically documented graft or native coronary artery occlusion or new severe stenosis with thrombosis and/or diminished epicardial flow, or (3) imaging evidence of new loss of viable myocardium or new regional wall motion abnormality. The endpoint of procedural myocardial infarction was included post hoc and the definition was adapted to match the definition applied in the EXCEL trial on PCI vs. CABG for LMCA stenosis. Peri-procedural MI due to repeat revascularization during follow-up were assessed applying the 3rd Universal definition as CK-MB was not available in all event patients. A procedural MI according to this definition was counted as a non-index procedural myocardial infarction.  **Target lesion revascularization:** Repeat revascularisation by PCI of any target segment treated during the index procedure. A target lesion segment was defined as a stented or balloon treated segment and its 5 mm margins.  **LMCA revascularization:** Any subsequent revascularisation by PCI of the segments within 5 mm of any treated segment related to the LMCA or the LMCA bifurcation. Any revascularisation by CABG of native LMCA including the LMCA bifurcation, or revascularisation of a graft supplying the left anterior descending artery or circumflex arteries.  **Definite stent thrombosis**: Stent thromboses were categorized as acute, subacute, late and very late and as definite, probable and possible according to ARC criteria. (Cutlip et al. Circulation 2007;115:2344–51)  **Symptomatic graft occlusion:** Diagnosis of symptomatic graft occlusion required it to be detected during a clinically indicated coronary angiography.  **Stroke:** Ischemic or haemorrhagic cerebrovascular event verified by brain computed tomography (CT) or magnetic resonance imaging (MRI).  **Pulmonary embolus:** The diagnosis of pulmonary embolus required verification by an appropriate computed tomography scan. |
| --- |
| **EXCEL (the Evaluation of XIENCE versus Coronary Artery Bypass Surgery for Effectiveness of Left Main Revascularization trial)**  **Death:** The cause of death will be adjudicated as being due to cardiovascular causes, non-cardiovascular causes, or undetermined causes.  Cardiovascular death includes sudden cardiac death, death due to acute MI, heart failure or cardiogenic shock, stroke, other cardiovascular causes, or bleeding  Non-cardiovascular death is defined as any death with known cause not of cardiac or vascular causes  Undetermined cause of death refers to a death not attributable to one of the above categories of cardiovascular death or to a noncardiovascular cause. For this trial all deaths of undetermined cause will be included in the cardiovascular category  **Myocardial infarction (protocol definition):**  **Post procedure MI:** Defined as the occurrence within 72 hours after either PCI or CABG of either:  CK-MB >10x upper reference limit (URL)*, OR  CK-MB >5x URL*, PLUS  -new pathological Q waves in at least 2 contiguous leads or new persistent non-rate related LBBB, or  -angiographically documented graft or native coronary artery occlusion or new severe stenosis with thrombosis and/or diminished epicardial flow, or  -imaging evidence of new loss of viable myocardium or new regional wall motion abnormality  **Spontaneous MI: d**efined as the occurrence >72 hours after any PCI or CABG of:  The rise and/or fall of cardiac biomarkers (CK-MB or troponin) >1x URL* PLUS:  - ECG changes indicative of new ischemia [ST -segment elevation or depression, in the absence of other causes of ST -segment changes such as left ventricular hypertrophy (LVH) or bundle branch block (BBB)], or  - Development of pathological Q waves (≥0.04 seconds in duration and ≥1 mm in depth) in ≥2 contiguous precordial leads or ≥2 adjacent limb leads) of the ECG, or  - Angiographically documented graft or native coronary artery occlusion or new severe stenosis with thrombosis and/or diminished epicardial flow, or  - Imaging evidence of new loss of viable myocardium or new regional wall motion abnormality  Each MI will also be adjudicated as:   ST-segment elevation MI (STEMI)   Non-ST-segment elevation MI (NSTEMI)   Each STEMI and NSTEMI will be subcategorized as  -Q-wave  -Non-Q-wave  -Unknown (no ECG or ECG not interpretable)  **Stroke:** The rapid onset of a new persistent neurologic deficit attributed to an obstruction in cerebral blood flow and/or cerebral hemorrhage with no apparent non-vascular cause (e.g., trauma, tumor, or infection). A vascular neurologist or stroke specialist will determine whether a stroke has occurred and determine the stroke severity using the NIHSS TIA/Stroke questionnaire. Available neuroimaging studies will be considered to support the clinical impression and to determine if there is a demonstrable lesion compatible with an acute stroke. Strokes will be classified as ischemic, hemorrhagic, or unknown. Four criteria must be fulfilled to diagnosis stroke:  1. Rapid onset of a focal/global neurological deficit with at least one of the following: change in level of consciousness, hemiplegia, hemiparesis, numbness or sensory loss affecting one side of the body, dysphasia/aphasia, hemianopia, amaurosis fugax, other new neurological sign(s)/symptom(s) consistent with stroke; and  2. Duration of a focal/global neurological deficit ≥24 hours or <24 hours if any of the following conditions exist:  i. At least one of the following therapeutic interventions:  a. Pharmacologic (i.e., thrombolytic drug administration)  b. Non-pharmacologic (i.e., neurointerventional procedure such as intracranial angioplasty)  ii. Available brain imaging clearly documents a new hemorrhage or infarct  iii. The neurological deficit results in death  3. No other readily identifiable non-stroke cause for the clinical presentation (e.g., brain tumor, trauma, infection, hypoglycemia, other metabolic abnormality, peripheral lesion, or drug side effect). Patients with non-focal global encephalopathy will not be reported as a stroke without unequivocal evidence based upon neuroimaging studies.  4. Confirmation of the diagnosis by a neurology or neurosurgical specialist and at least one of the following:  a. Brain imaging procedure (at least one of the following):  i. CT scan  ii. M RI scan  iii. Cerebral vessel angiography  b. Lumbar puncture (i.e. spinal fluid analysis diagnostic of intracranial hemorrhage)  All strokes with stroke disability of modified Rankin Scale (mRS) ≥1 (increase from baseline assessment) will be included in the primary endpoint. All diagnosed strokes (even with mRS 0) will also be tabulated.  **Ischemia-driven revascularization:**  A coronary revascularization procedure may be either a CABG or a PCI. The coronary segments revascularized will be sub-classified as:   Target Lesion: A lesion revascularized in the index procedure (or during a planned or provisional staged procedure). The LM target lesion extends from the left main stem ostium to the end of the 5 mm proximal segments of the left anterior descending and left circumflex arteries as well as the ramus intermedius if the latter vessel has a vessel diameter of ≥2 mm.   Target Vessel: The target vessel is defined as the entire major coronary vessel proximal and distal to the target lesion including upstream and downstream branches and the target lesion itself. The left main and any vessel originating from the left main coronary artery or its major branches is, by definition, considered a target vessel for the purposes of this trial (unless either the LAD or LCX are occluded at baseline and no attempt was made to revascularize these territories by either PCI or CABG).   Target Vessel Non-Target Lesion: The target vessel non-target lesion consists of a lesion in the epicardial vessel/branch/graft that contains the target lesion; however, this lesion is outside of the target lesion by at least 5 mm distal or proximal to the target lesion determined by quantitative coronary angiography (QCA).   Non-Target Vessel: For the purposes of this trial, the only possible non-target vessel would be the right coronary artery and its major branches that were not treated by either PCI or CABG at the index procedure (unless either the LAD or LCX are occluded at baseline and no attempt was made to revascularize these territories by either PCI or CABG).  All revascularization events will be adjudicated as either ischemia -driven or non-ischemia -driven. Revascularization will be considered ischemia driven if the diameter stenosis of the revascularized coronary segment is ≥50% by QCA and any of the following criteria for ischemia are met:  •A positive functional study corresponding to the area served by the target lesion; or  •Ischemic ECG changes at rest in a distribution consistent with the target vessel; or  •Typical ischemic symptoms referable to the target lesion; or  •IVUS of the target lesion with a minimal lumen area (MLA) of ≤4 mm^2^ for non-left main lesions or ≤6 mm^2^ for left main lesions. If the lesions are de novo (i.e. not restenotic), the plaque burden must also be ≥60%; or  •FFR of the target lesion ≤0.80  A target lesion revascularization for a diameter stenosis less than 50% might also be considered ischemia-driven by the Clinical Events Committee if there was a markedly positive functional study or ECG changes corresponding to the area served by the target lesion.  **Peri-procedural major adverse events:**  The composite rate of any of the following, occurring within 30 -days post procedure  •Death  •Stroke  •Myocardial infarction  •Ischemia -driven revascularization  •TIMI major or minor bleeding  •Transfusion ≥2 units of blood  •Major arrhythmia (supraventricular tachycardia requiring cardioversion, ventricular tachycardia or fibrillation requiring treatment, or bradyarrhythmia requiring temporary or permanent pacemaker)  •Any unplanned surgery or therapeutic radiologic procedure  •Renal failure (serum creatinine increase by ≥0.5 mg/dL from baseline or need for dialysis )  •Sternal wound dehiscence  •Infection requiring antibiotics  •Prolonged intubation (>48 hours)  •Post-pericardiotomy syndrome |
| **LE MANS (Study of UnprotectedLeft Main Stenting Versus Bypass Surgery)**  The major adverse events (MAE) were defined as all-cause mortality, acute myocardial infarction (defined as an increase in creatine phosphokinase (CPK)-MB to higher than 3 times the upper limit of normal after PCI and 5 times after CABG), repeat revascularization, acute heart failure (e.g., pulmonary edema, cardiogenic shock), or low output syndrome requiring intravenous inotropic agents and/or intra-aortic balloon pump support, post-procedural complications leading to reintervention, stroke, arrhythmia (ventricular fibrillation, ventricular tachycardia, or atrial fibrillation), major bleeding requiring additional blood transfusion, and infections compromising post-procedural rehabilitation. Any cardiac mortality, acute myocardial infarction, stroke, repeat intervention, and/or acute/subacute in-stent thrombosis were considered MACCE.  Target vessel failure was defined as any MACCE related to insufficient flow through the LMCA, and TVR as any repeat intervention (PCI or CABG) caused by a narrowing of the LMCA. The incidence of stent thrombosis was evaluated in accordance with the Academic Research Consortium Definitions of Stent Thrombosis |
| **PRECOMBAT (the Premier of Randomized Comparison of Bypass Surgery versus Angioplasty Using Sirolimus-Eluting Stent in Patients with Left Main Coronary Artery Disease trial)**  **Deaths** were considered cardiac unless an unequivocal, noncardiac cause was established.  **MI** was defined as new Q waves and increase in the creatine kinase MB concentration to greater than five times the upper limit of the normal range, if occurring within 48 hours after the procedure, or as new Q waves or an increase increatine kinase MB concentration to greater than the upper limit of the normal range, plus ischemic symptoms or signs, if occurring more than 48 hours after the procedure.  **Stroke** was defined as a sudden onset of vertigo, numbness, aphasia, or dysarthria resulting from vascular lesions of the brain, including hemorrhage, embolism, thrombosis, or rupturing aneurysm, and persisting for >24 hours.  **Protocol-defined graft occlusion or stent thrombosis** was defined as clinical presentation of an acute coronary syndrome with documentation of flow limiting thrombus or occlusion within a bypass graft or adjacent to the anastomosis of a previously bypassed coronary artery (for CABG patients), or within or adjacent to a previously successfully treated artery (for PCI patients), or as a Q wave in the territory of 1 treated vessels within 30 days.  **Target vessel revascularization (TVR)**, in which repeat revascularization witheither PCI or CABG was performed in the treated vessel, was considered to be driven by ischemia if the stenosis of any vessel was at least 50% of the diameter of the vessel in the presence of ischemic signs or symptoms or if the stenosis was at least 70% of the diameter of the vessel even in the absence of ischemic signs or symptoms. Alternatively, TVR was considered clinically driven when the treated vessels had stenosis of at least 50% in the presence of ischemic signs or symptoms. In addition, any revascularization was defined as revascularization in any vessel during followup.  **Crossover** was defined as completion of the procedure opposite the one designated by the randomization scheme within 30 days after randomization. |
| **SYNTAX (The other Synergy between Percutaneous Coronary Intervention with Taxus and Cardiac Surgery trial)**  **Deaths** were considered cardiac unless an unequivocal, noncardiac cause was established.  **CVA** was defined as a focal, central neurological deficit lasting >72 hours (h) which resulted in irreversible brain damage or body impairment.  **Repeat revascularization** was defined as any repeat PCI or CABG. Complete revascularization was defined as the successful treatment of all eligible lesions identified during the Heart Team conference and estimated post procedure by the investigator.  **MI** was based on previous studies, MI was defined in relation to intervention status as follows i) after allocation but before treatment: Q -wave (new pathological Q-waves in ≥2 leads lasting ≥0.04 seconds with CK-MB levels elevated above normal), and non-Q wave MI (elevation of CK levels >2 times the upper limit of normal [ULN] with positive CK-MB or elevation of CK levels to >2 times ULN without new Q-waves if no baseline CK-MB was available); ii) <7d after intervention: new Q-waves and either peak CK-MB/total CK >10% or plasma level of CK-MB 5x ULN; iii) 7d after intervention: new Q waves or peak CK-MB/total CK >10% or plasma level of CK-MB 5x ULN or plasma level of CK 5x ULN. The CK/CK-MB enzyme levels were obtained and measured by a core laboratory for all randomized patients.  **Per protocol graft occlusion (GO) and stent thrombosis (ST)** were considered acute if occurring ≤24h following the study procedure, sub-acute if occurring >24h to ≤30d following the study procedure and late after 30d. Per protocol graft occlusion and stent thrombosis were defined as either: i) clinical presentation of an acute coronary syndrome with documentation of a flow limiting thrombus or occlusion within a bypass graft or adjacent to the anastomosis of a previously bypassed coronary artery (for CABG patients) or within or adjacent to a previously successfully treated artery (for PCI patients); ii) a Q-wave MI in the territory of ≥1 treated vessels within first 30 days (d). |
| **Boudriot, et al**  Myocardial infarction was defined as an increase increatine kinase-MB activity >3 times the upper limit of normal after PCI and >5 times after CABG. In addition, standard electrocardiographic criteria were applied.  The incidence of stent thrombosis was evaluated in accordance with the Academic Research Consortium definitions.  Repeat revascularization was defined as anyrevascularization by CABG or PCI within 12 months, and was subdivided into target lesion revascularization of the ULM and distally located lesions or those of the right coronary artery. |

**
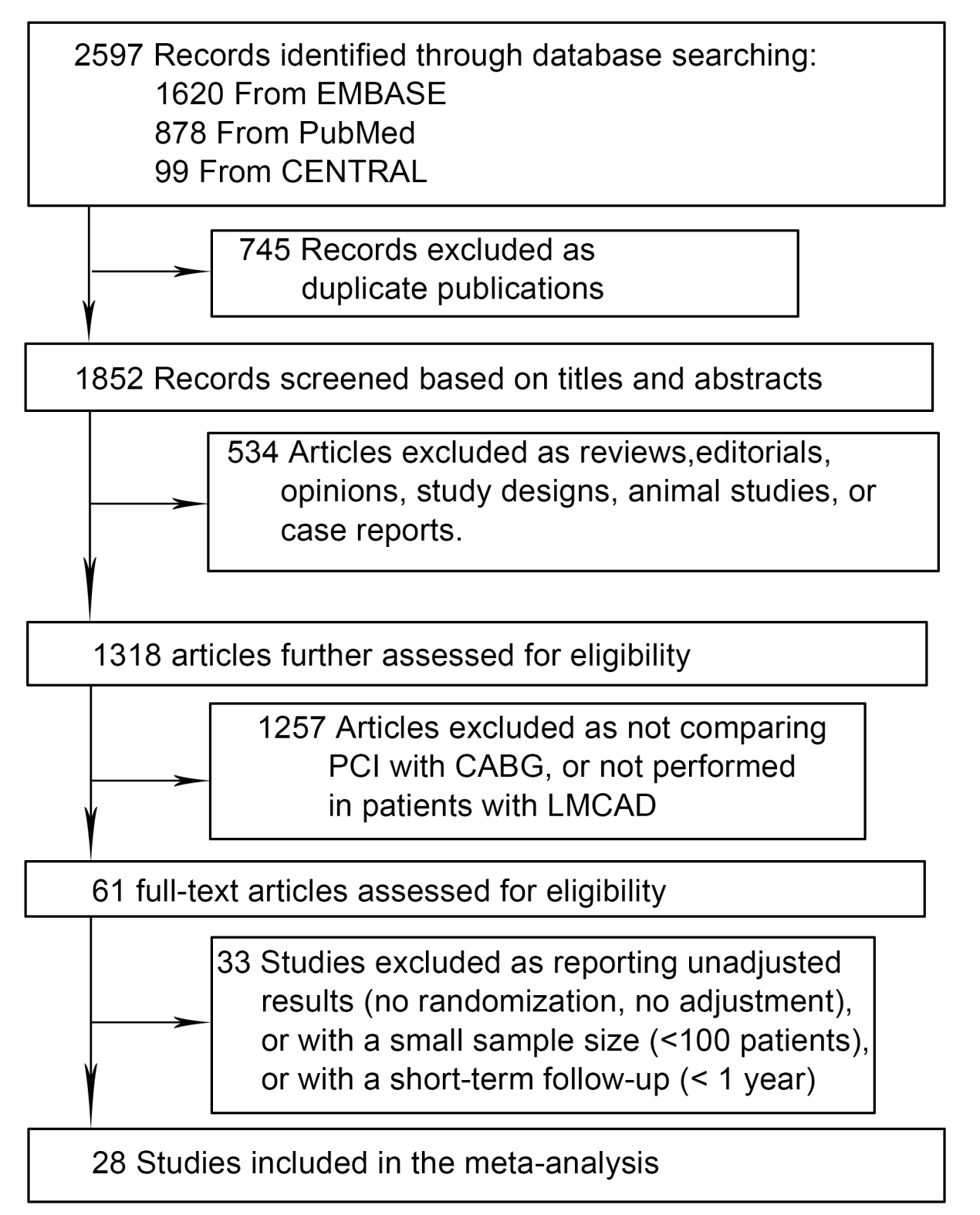
**

**Figure S1.** Flow diagram of study selection. CABG = coronary artery bypass graft surgery; CENTRAL = the Cochrane Central Register of Controlled Trials; LMCAD = left main coronary artery disease; PCI = percutaneous coronary intervention.

**Table S2.** Risk of bias of included randomized controlled trials.

| **NOBLE** |  |  |
| --- | --- | --- |
| **Risk of bias** | **Authors’ judgement** | **Support for judgement** |
| Random sequence generation (selection bias) | Low risk | Computer-generated using permuted random blocks |
| Allocation concealment (selection bias) | Low risk | Web-based computer randomization |
| Blinding of participants and personnel (performance bias) | High risk | Blinding not applicable |
| Blinding of outcome assessment (detection bias) | Low risk | Outcome assessors blinded |
| Incomplete outcome data (attrition bias) | Unclear risk | Over 20% losses (31% losses to follow-up) |
| Selective reporting (reporting bias) | Low risk | All expected outcomes included |
| Other bias | Unclear risk | the primary endpoint timing changed |
| **EXCEL** |  |  |
| **Risk of bias** | **Authors’ judgement** | **Support for judgement** |
| Random sequence generation (selection bias) | Low risk | Variable block random allocation |
| Allocation concealment (selection bias) | Low risk | Interactive voice-based or Web-based system |
| Blinding of participants and personnel (performance bias) | High risk | Blinding not applicable |
| Blinding of outcome assessment (detection bias) | Low risk | Outcome assessors blinded |
| Incomplete outcome data (attrition bias) | Low risk | 8% of participants were lost to follow-up; reasons reported |
| Selective reporting (reporting bias) | Low risk | Study protocol is available, all expected outcomes included |
| Other bias | Low risk | Free of other sources of bias |
| **LE MANS** |  |  |
| **Risk of bias** | **Authors’ judgement** | **Support for judgement** |
| Random sequence generation (selection bias) | Unclear risk | Randomization stated to have been done but no method reported |
| Allocation concealment (selection bias) | Unclear risk | Not specified |
| Blinding of participants and personnel (performance bias) | High risk | Blinding not applicable |
| Blinding of outcome assessment (detection bias) | Low risk | Blinded outcome assessors |
| Incomplete outcome data (attrition bias) | Low risk | 11.4% of participants were lost to follow-up; reasons reported |
| Selective reporting (reporting bias) | Low risk | Include all expected outcomes |
| Other bias | Low risk | Free of other sources of bias |
| **PRECOMBAT** |  |  |
| **Risk of bias** | **Authors’ judgement** | **Support for judgement** |
| Random sequence generation (selection bias) | Low risk | Computer-generated random allocation sequence |
| Allocation concealment (selection bias) | Low risk | Sealed envelopes concealed the allocation |
| Blinding of participants and personnel (performance bias) | High risk | Blinding not applicable |
| Blinding of outcome assessment (detection bias) | Low risk | Outcome assessors blinded |
| Incomplete outcome data (attrition bias) | Low risk | 6.8% of participants were lost to follow-up; reasons reported |
| Selective reporting (reporting bias) | Low risk | Study protocol is available, all expected outcomes included |
| Other bias | Low risk | Free of other sources of bias |
| **SYNTAX** |  |  |
| **Risk of bias** | **Authors’ judgement** | **Support for judgement** |
| Random sequence generation (selection bias) | Low risk | Variable block random allocation |
| Allocation concealment (selection bias) | Low risk | Central allocation (Interactive Voice Response System) |
| Blinding of participants and personnel (performance bias) | High risk | Blinding not applicable |
| Blinding of outcome assessment (detection bias) | Low risk | Outcome assessors blinded |
| Incomplete outcome data (attrition bias) | Low risk | 5.2% of participants were lost to follow-up; reasons reported |
| Selective reporting (reporting bias) | Low risk | Study protocol is available, all expected outcomes included |
| Other bias | Low risk | Free of other sources of bias |
| **Boudriot, et al** |  |  |
| **Risk of bias** | **Authors’ judgement** | **Support for judgement** |
| Random sequence generation (selection bias) | Low risk | Computerized randomization program |
| Allocation concealment (selection bias) | Unclear risk | Concealment of allocation was not reported |
| Blinding of participants and personnel (performance bias) | High risk | Blinding not applicable |
| Blinding of outcome assessment (detection bias) | Low risk | Blinded outcome assessors |
| Incomplete outcome data (attrition bias) | Low risk | 0.5% of participants were lost to follow-up |
| Selective reporting (reporting bias) | Low risk | All expected outcomes included |
| Other bias | Low risk | Free of other sources of bias |

Expanded study abbreviations are as follows: EXCEL = the Evaluation of XIENCE versus Coronary Artery Bypass Surgery for Effectiveness of Left Main Revascularization trial; LE MANS = Study of UnprotectedLeft Main Stenting Versus Bypass Surgery; NOBLE = The Nordic-Baltic-British left main revascularisation study; PRECOMBAT = the Premier of Randomized Comparison of Bypass Surgery versus Angioplasty Using Sirolimus-Eluting Stent in Patients with Left Main Coronary Artery Disease trial; SYNTAX = the other Synergy between Percutaneous Coronary Intervention with Taxus and Cardiac Surgery trial.

**Table S3.** Study quality of included matched observational studies using the Newcastle-Ottawa scale.

| **Zheng, et al** |  |  |
| --- | --- | --- |
| **NOS criteria** | **Authors’ judgement** | **Support for judgement** |
| Selection (4 points) | 4 | Consecutive patients truly representative of the cohort; ascertainment of exposure to implants with secure records; outcomes of interest were not present at start of study |
| Comparability (2 points) | 2 | Propensity score adjustment controlling for 15 important confounding factors |
| Outcome (3 points) | 3 | Assessment of outcome adjudicated independently by an events committee; follow up were adequate and long enough (3 years) |
| **Yu, et al** |  |  |
| **NOS criteria** | **Authors’ judgement** | **Support for judgement** |
| Selection (4 points) | 4 | Consecutive patients truly representative of the cohort; ascertainment of exposure to implants with secure records; outcomes of interest were not present at start of study |
| Comparability (2 points) | 2 | Propensity score adjustment controlling for 18 important confounding factors |
| Outcome (3 points) | 3 | Assessment of outcome assigned by the patients’ physicians and adjudicated independently by an events committee; follow up were adequate (93.2%) and long enough (7.1 years) |
| **Lu, et al** |  |  |
| **NOS criteria** | **Authors’ judgement** | **Support for judgement** |
| Selection (4 points) | 4 | Consecutive patients truly representative of the cohort; ascertainment of exposure to implants with secure records; outcomes of interest were not present at start of study |
| Comparability (2 points) | 2 | Propensity score adjustment controlling for a number of important confounding factors |
| Outcome (3 points) | 2 | Assessment of outcome not reported; follow up were adequate (100%) and long enough (4.3 years) |
| **Wei, et al** |  |  |
| **NOS criteria** | **Authors’ judgement** | **Support for judgement** |
| Selection (4 points) | 4 | Consecutive patients truly representative of the cohort; ascertainment of exposure to implants with secure records; outcomes of interest were not present at start of study |
| Comparability (2 points) | 2 | Multivariate adjustment performed |
| Outcome (3 points) | 1 | Assessment of outcome not reported; follow up were adequate (100%), but might not be long enough (1.3 years) |
| **IRIS-MAIN registry-1** |  |  |
| **NOS criteria** | **Authors’ judgement** | **Support for judgement** |
| Selection (4 points) | 4 | Consecutive patients truly representative of the cohort; ascertainment of exposure to implants with secure records; outcomes of interest were not present at start of study |
| Comparability (2 points) | 2 | Multivariate adjustment performed for a number of important factors |
| Outcome (3 points) | 3 | Assessment of outcome assigned by independent research personnel; follow up were adequate and long enough (9.7 years) |
| **IRIS-MAIN registry-2** |  |  |
| **NOS criteria** | **Authors’ judgement** | **Support for judgement** |
| Selection (4 points) | 4 | Consecutive patients truly representative of the cohort; ascertainment of exposure to implants with secure records; outcomes of interest were not present at start of study |
| Comparability (2 points) | 2 | Multivariate adjustment performed for a number of important factors |
| Outcome (3 points) | 3 | Assessment of outcome assigned by independent research personnel; follow up were adequate and long enough (5.6 years) |
| **IRIS-MAIN registry-3** |  |  |
| **NOS criteria** | **Authors’ judgement** | **Support for judgement** |
| Selection (4 points) | 4 | Consecutive patients truly representative of the cohort; ascertainment of exposure to implants with secure records; outcomes of interest were not present at start of study |
| Comparability (2 points) | 2 | Multivariate adjustment performed for a number of important factors |
| Outcome (3 points) | 3 | Assessment of outcome assigned by independent research personnel; follow up were adequate and long enough (3 years) |
| **Jeong, et al** |  |  |
| **NOS criteria** | **Authors’ judgement** | **Support for judgement** |
| Selection (4 points) | 4 | Consecutive patients somewhat representative of the cohort; ascertainment of exposure to implants with secure records; outcomes of interest were not present at start of study |
| Comparability (2 points) | 2 | Propensity-matched population |
| Outcome (3 points) | 2 | Assessment of outcome not reported; follow up were adequate and long enough (4.7 years) |
| **DELTA registry** |  |  |
| **NOS criteria** | **Authors’ judgement** | **Support for judgement** |
| Selection (4 points) | 4 | Consecutive patients somewhat representative of the cohort; ascertainment of exposure to implants with secure records; outcomes of interest were not present at start of study |
| Comparability (2 points) | 2 | Propensity-matched population |
| Outcome (3 points) | 3 | Assessment of outcome with record linkage; follow up were adequate (95%) and long enough (3.5 years) |
| **CREDO-Kyoto 2** |  |  |
| **NOS criteria** | **Authors’ judgement** | **Support for judgement** |
| Selection (4 points) | 4 | Consecutive patients truly representative of the cohort; ascertainment of exposure to implants with secure records; outcomes of interest were not present at start of study |
| Comparability (2 points) | 2 | Multivariate adjustment performed for 30 clinically relevant factors |
| Outcome (3 points) | 3 | Assessment of outcome adjudicated by the clinical event committee; follow up were adequate and long enough (3 years) |
| **Chang, et al** |  |  |
| **NOS criteria** | **Authors’ judgement** | **Support for judgement** |
| Selection (4 points) | 4 | Consecutive patients somewhat representative of the cohort; ascertainment of exposure to implants with secure records; outcomes of interest were not present at start of study |
| Comparability (2 points) | 2 | Propensity-matched population |
| Outcome (3 points) | 3 | Assessment of outcome adjudicated by and independent group of clinicians; follow up were adequate (98.9%) and long enough (4.2 years) |
| **Yi, et al** |  |  |
| **NOS criteria** | **Authors’ judgement** | **Support for judgement** |
| Selection (4 points) | 4 | Consecutive patients somewhat representative of the cohort; ascertainment of exposure to implants with secure records; outcomes of interest were not present at start of study |
| Comparability (2 points) | 2 | Propensity-matched population |
| Outcome (3 points) | 2 | Assessment of outcome not reported; follow up were adequate (97.7%) and long enough (5 years) |
| **CUSTOMIZE registry** |  |  |
| **NOS criteria** | **Authors’ judgement** | **Support for judgement** |
| Selection (4 points) | 4 | Consecutive patients somewhat representative of the cohort; ascertainment of exposure to implants with secure records; outcomes of interest were not present at start of study |
| Comparability (2 points) | 2 | Propensity-matched population |
| Outcome (3 points) | 2 | Assessment of outcome adjudicated independently by an events committee; follow up were long enough (3 years) |
| **Rittger, et al** |  |  |
| **NOS criteria** | **Authors’ judgement** | **Support for judgement** |
| Selection (4 points) | 4 | Consecutive patients truly representative of the cohort; ascertainment of exposure to implants with secure records; outcomes of interest were not present at start of study |
| Comparability (2 points) | 2 | Propensity score adjustment controlling for 9 important confounding factors |
| Outcome (3 points) | 1 | Assessment of outcome not reported; follow up were adequate and might not be long enough (1 year) |
| **Asan-Multivessel Registry** |  |  |
| **NOS criteria** | **Authors’ judgement** | **Support for judgement** |
| Selection (4 points) | 4 | Consecutive patients truly representative of the cohort; ascertainment of exposure to implants with secure records; outcomes of interest were not present at start of study |
| Comparability (2 points) | 2 | Propensity score adjustment controlling for a number of important confounding factors |
| Outcome (3 points) | 3 | Assessment of outcome verified and adjudicated by independent clinicians; follow up were adequate (97.4%) and long enough (5.6 years) |
| **Kang, et al** |  |  |
| **NOS criteria** | **Authors’ judgement** | **Support for judgement** |
| Selection (4 points) | 4 | Consecutive patients somewhat representative of the cohort; ascertainment of exposure to implants with secure records; outcomes of interest were not present at start of study |
| Comparability (2 points) | 2 | Propensity-matched population |
| Outcome (3 points) | 1 | Assessment of outcome not reported; follow up were adequate but might not be long enough (2.8 years) |
| **Mäkikallio, et al** |  |  |
| **NOS criteria** | **Authors’ judgement** | **Support for judgement** |
| Selection (4 points) | 4 | Consecutive patients truly representative of the cohort; ascertainment of exposure to implants with secure records; outcomes of interest were not present at start of study |
| Comparability (2 points) | 2 | Multivariate adjustment performed for clinically relevant factors |
| Outcome (3 points) | 1 | Assessment of outcome not reported; follow up were adequate and but might not be long enough (1 year) |
| **MAIN-COMPARE registry** |  |  |
| **NOS criteria** | **Authors’ judgement** | **Support for judgement** |
| Selection (4 points) | 4 | Consecutive patients somewhat representative of the cohort; ascertainment of exposure to implants with secure records; outcomes of interest were not present at start of study |
| Comparability (2 points) | 2 | Propensity-matched population |
| Outcome (3 points) | 3 | Assessment of outcome assigned by the patient’s physician and centrally adjudicated by an independent group of clinicians; follow up were adequate (98.9%) and long enough (3 years) |
| **Ghenim, et al** |  |  |
| **NOS criteria** | **Authors’ judgement** | **Support for judgement** |
| Selection (4 points) | 4 | Consecutive patients truly representative of the cohort; ascertainment of exposure to implants with secure records; outcomes of interest were not present at start of study |
| Comparability (2 points) | 2 | Propensity score adjustment controlling for a number of important confounding factors |
| Outcome (3 points) | 1 | Assessment of outcome not reported; follow up were adequate and but might not be long enough (1 year) |
| **White, et al** |  |  |
| **NOS criteria** | **Authors’ judgement** | **Support for judgement** |
| Selection (4 points) | 4 | Consecutive patients truly representative of the cohort; ascertainment of exposure to implants with secure records; outcomes of interest were not present at start of study |
| Comparability (2 points) | 2 | Propensity score adjustment controlling for 18 important confounding factors |
| Outcome (3 points) | 2 | Assessment of outcome with record linkage; follow up were adequate and but might not be long enough (2 years) |
| **Rodes-Cabau, et al** |  |  |
| **NOS criteria** | **Authors’ judgement** | **Support for judgement** |
| Selection (4 points) | 4 | Consecutive patients truly representative of the cohort; ascertainment of exposure to implants with secure records; all outcomes of interest were not present at start of study |
| Comparability (2 points) | 2 | Propensity score adjustment controlling for a number of important confounding factors |
| Outcome (3 points) | 2 | Assessment of outcome with record linkage; follow up were adequate and but might not be long enough (2 years) |
| **Palmerini, et al** |  |  |
| **NOS criteria** | **Authors’ judgement** | **Support for judgement** |
| Selection (4 points) | 4 | Consecutive patients truly representative of the cohort; ascertainment of exposure to implants with secure records; outcomes of interest were not present at start of study |
| Comparability (2 points) | 2 | Propensity score adjustment controlling for a number of important confounding factors |
| Outcome (3 points) | 2 | Assessment of outcome with record linkage; follow up were adequate and but might not be long enough (2 years) |

Expanded study abbreviations are as follows: CREDO-Kyoto 2 = the Coronary Revascularization Demonstrating Outcome Study in Kyoto (CREDO-Kyoto) PCI/CABG Registry Cohort-2; CUSTOMIZE = the Appraise a Customized Strategy for Left Main Revascularization Registry; DELTA = the drug-eluting stent for left main coronary artery disease registry; IRIS-MAIN = Interventional Research Incorporation Society-Left MAIN Revascularization registry; MAIN-COMPARE = Revascularization for Unprotected Left Main Coronary Artery Stenosis: Comparison of Percutaneous Coronary Angioplasty versus Surgical Revascularization registry.

**Table S4.** Other selected baseline characteristics of comparative randomized controlled trials and matched observational studies.

| Trial | Year | EuroSCORE (PCI) | EuroSCORE (CABG) | SYNTAX (PCI) | SYNTAX (CABG) | Not isolated LM, % | Distal LM, % |
| --- | --- | --- | --- | --- | --- | --- | --- |
| NOBLE | 2016 | 2 (2, 4) | 2 (2, 4) | 22.5±7.5 | 22.4±8.0 | NA | 81 |
| EXCEL | 2016 | NA | NA | 26.9±8.8 | 26.0±9.8 | 82.2 | 79.2 |
| LE MANS | 2016 | 3.3±2.3 | 3.5±2.3 | 25.2±8.7 | 24.7±6.8 | 94 | 60 |
| PRECOMBAT | 2015 | 2.6±1.8 | 2.8±1.9 | 24.4±9.4 | 25.8±10.5 | NA | 67 |
| SYNTAX | 2014 | 3.9±2.8 | 3.9±2.9 | 29.6±13.5 | 30.2±12.7 | 85.9 | 58.3 |
| Boudriot, et al | 2011 | 2.4 (1.5, 3.7) | 2.6 (1.7, 4.9) | 24.0 (19.0, 29.0) | 23.0 (14.8, 28.0) | 71 | 69 |
| Zheng, et al | 2016 | 1.8±1.8 | 2.8±2.1 | 23.6±6.7 | 33.3±7.8 | 95 | NA |
| Yu, et al | 2016 | 5 (3, 6) | 5 (3, 6) | NA | NA | 93.7% | 68 |
| Lu, et al | 2016 | 7.1±5.1 | 6.4±4.0 | NA | NA | 98 | NA |
| Wei, et al | 2016 | 6.8±0.22 | 6.0±0.37 | 27.3±6.9 | 35.7±6.2 | 97 | NA |
| IRIS-MAIN registry-1 | 2016 | NA | NA | NA | NA |  |  |
| IRIS-MAIN registry-2 | 2016 | NA | NA | NA | NA |  |  |
| IRIS-MAIN registry-3 | 2016 | NA | NA | NA | NA |  |  |
| Jeong, et al | 2013 | NA | NA | 24±8 | 26±7 | NA | NA |
| DELTA registry | 2012 | 5.0±3.6 | 5.1±2.9 | NA | NA | 93.5 | 64.8 |
| CREDO-Kyoto 2 | 2012 | NA | NA | 26.5 (21, 34) | 30 (22, 40) | 91 | NA |
| Chang, et al | 2012 | 4.32±2.44 | 4.05±2.39 | 30.62±11.10 | 32.35±13.18 | 92 | NA |
| Yi, et al | 2012 | NA | NA | NA | NA | 79.7 | NA |
| CUSTOMIZE registry | 2011 | NA | NA | 21.6±5.8 | 21.5±6.3 | NA | 65.9 |
| Rittger, et al | 2011 | 2.9±2.7 | 4±4.3 | NA | NA | 67 | NA |
| Asan-Multivessel Registry | 2011 | NA | NA | NA | NA | NA | NA |
| Kang, et al | 2010 | 3.6±3.0 | 4.8±4.1 | NA | NA | 89.5 | 74.3 |
| Mäkikallio, et al | 2009 | 7.7±7.5 | 5.2±4.4 | NA | NA | NA | 80 |
| MAIN-COMPARE registry | 2008 | NA | NA | NA | NA | 88.9 | 52.2 |
| Ghenim, et al | 2009 | 8 (6, 9) | 7 (6, 8) | NA | NA | 95.3 | 75.5 |
| White, et al | 2008 | NA | NA | NA | NA | NA | NA |
| Rodes-Cabau, et al | 2008 | 8.4±2.5 | 9.5±2.6 | NA | NA | NA | 60 |
| Palmerini, et al | 2007 | 8 (4, 18) | 7 (3, 14) | NA | NA | 74 | NA |

Values are mean, median (interquartile range), or %. CABG = coronary artery bypass graft surgery; EF = ejection fraction; FU = follow-up; LM = left main coronary; MI = myocardial infarction; NA = not available; PCI = percutaneous coronary intervention.

Expanded study abbreviations are as follows: CUSTOMIZE = the Appraise a Customized Strategy for Left Main Revascularization Registry; DELTA = the drug-eluting stent for left main coronary artery disease registry; EXCEL = the Evaluation of XIENCE versus Coronary Artery Bypass Surgery for Effectiveness of Left Main Revascularization trial; IRIS-MAIN = Interventional Research Incorporation Society-Left MAIN Revascularization registry; LE MANS = Study of UnprotectedLeft Main Stenting Versus Bypass Surgery; MAIN-COMPARE = Revascularization for Unprotected Left Main Coronary Artery Stenosis: Comparison of Percutaneous Coronary Angioplasty versus Surgical Revascularization registry; NOBLE = The Nordic-Baltic-British left main revascularisation study; PRECOMBAT = the Premier of Randomized Comparison of Bypass Surgery versus Angioplasty Using Sirolimus-Eluting Stent in Patients with Left Main Coronary Artery Disease trial; SYNTAX = the other Synergy between Percutaneous Coronary Intervention with Taxus and Cardiac Surgery trial. CREDO-Kyoto 2 = the Coronary Revascularization Demonstrating Outcome Study in Kyoto (CREDO-Kyoto) PCI/CABG Registry Cohort-2.

**Table S5.** Main inclusion and exclusion criteria, primary and secondary endpoints of randomized controlled trials.

| **Trial** | **Main inclusion criteria** | **Main exclusion criteria** | **Primary endpoint** | **Secondary endpoint** |
| --- | --- | --- | --- | --- |
| NOBLE | stable angina pectoris, unstable angina pectoris, or acute coronary syndrome, together with a lesion with visually assessed stenosis diameter ≥50% or fractional flow reserve ≤0·80 in the left main coronary artery ostium, mid-shaft, or bifurcation, with no more than three additional noncomplex lesions. | ST-elevation infarction within 24 h, being considered too high risk for CABG or PCI, or expected survival of less than 1 year. | Composite of major adverse cardiac and cerebrovascular events (MACCE; death from any cause, non-procedural myocardial infarction, repeat revascularisation, or stroke). | The individual component of the primary MACCE endpoint, definite stent thrombosis, and symptomatic graft occlusion. Procedural myocardial infarctions were documented (post hoc). Repeat revascularisations. |
| EXCEL | Stenosis of the left main coronary artery of 70% or more, as estimated visually, or stenosis of 50% to less than 70% if determined by means of noninvasive or invasive testing to be hemodynamically significant, and a consensus among the members of the heart team regarding eligibility for revascularization with either PCI or CABG. In addition, participants were required to have low-to-intermediate anatomical complexity of coronary artery disease, as defined by a site-determined SYNTAX score of 32 or lower (the SYNTAX score reflects a comprehensive angiographic assessment of the coronary vasculature, with 0 as the lowest score and higher scores [no upper limit] indicating more complex coronary anatomy). | Prior PCI of the left main trunk at any time prior to randomization, PCI of any other (non-left main) coronary artery lesions within one year prior to randomization, CABG at any time prior to randomization. Need for any concomitant cardiac surgery other than CABG.  Angiographic exclusion criteria: a. Left main diameter stenosis <50%, unless left main equivalent disease is present; b. SYNTAX score ≥33, as determined by the local Heart Team; c. Visually estimated left main reference vessel diameter <2.25 mm or >4.25 mm  d. The presence of specific coronary lesion characteristics or other cardiac condition(s) which leads the participating interventional cardiologist or cardiac surgeon to believe that clinical equipoise is not present | the primary composite end point of death from any cause, stroke, or myocardial infarction | a composite of death from any cause, stroke, or myocardial infarction at 30 days and the rate of a composite of death, stroke, myocardial infarction, or ischemia-driven revascularization at 3 years. Additional secondary end points included the components of the primary end point, as well as revascularization, stent thrombosis, symptomatic graft stenosis or occlusion, bleeding complications, and a prespecified composite of periprocedural major adverse events. |
| LE MANS | Patients with >50% narrowing of ULMCA, with or without multivessel coronary artery disease suitable for equal revascularization both with PCI and CABG. All patients had to be symptomatic with documented myocardial ischemia. | Acute myocardial infarction, total occlusion of left main, comorbid conditions, or coronary anatomic considerationsthat increased the surgical risk to a Euroscore of 8 or more, stroke or transient ischemic attack within 3 months, renal dysfunction, or contraindication to antiplatelet therapy. | The change in LVEF assessed by 2-dimensional echocardiography 12 months | MAE and MACCE, length of hospitalization, exercise tolerance measured with an electrocardio graphic treadmill stress test along with angina severity according to the Canadian Cardiovascular Society classification after 1 year, total survival and freedom from MACCE, and target vessel failure (TVF) and revascularization (TVR). |
| PRECOMBAT | Older than 18 years of age and had received a diagnosis of stable angina, unstable angina, silent ischemia, or non–ST-segment elevation MI. All patients had newly diagnosed ULMCA stenosis (more than 50% diameter stenosis by visual angiographic estimation) and had been judged to be suitable candidates for either PCI or CABG. | Systemic (intravenous) sirolimus use within 12 months. Any previous percutaneous coronary intervention (PCI) within 1 year. Previous bypass surgery. Any previous PCI of a ULMCA or ostial left circumflex artery or ostial left anterior descending artery lesion within 1 year. Acute MI within 1 week. Ejection fraction <30%. Cardiogenic shock. | Composite of death from any cause, MI, stroke, or ischemia-driven target vessel revascularization [TVR]) | The individual components of the primary endpoint; a composite of death, MI, or stroke; and clinically driven TVR. |
| SYNTAX | De novo lesions, ≥50% target vessel stenosis with stable/unstable angina or atypical chest pain. If asymptomatic, positive evidence of myocardial ischemia was required. | Previous PCI or CABG, acute myocardial infarction (MI), or the need for concomitant cardiac surgery. | Composite of major adverse cardiac and cerebrovascular events (i.e., death from any cause, stroke, myocardial infarction, or repeat revascularization) | The individual component of the primary MACCE endpoint, Quality of life and costeffectiveness. |
| Boudriot, et al | Patients age 18 to 80 years with stenosis (>50%) of the ULM with or without additional multivessel coronary artery disease were included in this multicenter study. Patients had to be symptomatic or have documented myocardial ischemia. | Myocardial infarction48 h requiring immediate intervention, additional valvular heart disease requiring surgery, previous surgical treatment for coronary artery or valvular disease, severe peripheral arterial disease, significant carotid stenosis requiring treatment, renal dysfunction requiring dialysis, any disease with limited life expectancy, overt congestive heart failure, and contraindication to antiplatelet therapy. Angiographic exclusion criteria were total occlusions, extreme left-dominant coronary artery perfusion, and distal lesion length >30 mm in a single lesion | Major adverse cardiovascular events, which included death from any cause, myocardial infarction, and the need for repeat revascularization | Each individual component of the composite end point. |

Expanded study abbreviations are as follows: EXCEL = the Evaluation of XIENCE versus Coronary Artery Bypass Surgery for Effectiveness of Left Main Revascularization trial; LE MANS = Study of UnprotectedLeft Main Stenting Versus Bypass Surgery; NOBLE = The Nordic-Baltic-British left main revascularisation study; PRECOMBAT = the Premier of Randomized Comparison of Bypass Surgery versus Angioplasty Using Sirolimus-Eluting Stent in Patients with Left Main Coronary Artery Disease trial; SYNTAX = the other Synergy between Percutaneous Coronary Intervention with Taxus and Cardiac Surgery trial.


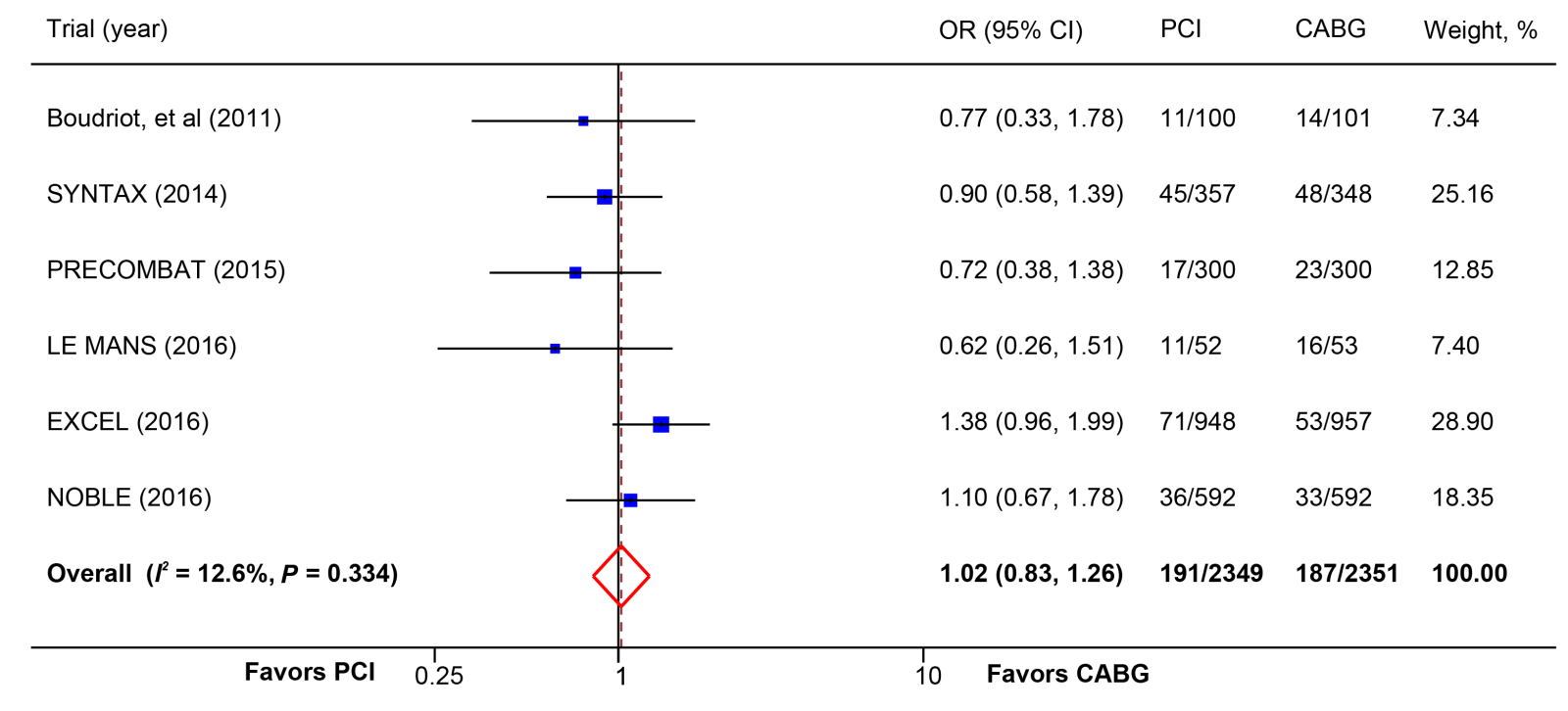


**Figure S2.** Pooled odds ratio for total mortality with percutaneous coronary intervention versus coronary artery bypass graft surgery.

CABG = coronary artery bypass graft surgery; CI = confidence interval; OR = odds ratio; PCI = percutaneous coronary intervention. Expanded study abbreviations are as follows: EXCEL = the Evaluation of XIENCE versus Coronary Artery Bypass Surgery for Effectiveness of Left Main Revascularization trial; LE MANS = Study of UnprotectedLeft Main Stenting Versus Bypass Surgery; NOBLE = The Nordic-Baltic-British left main revascularisation study; PRECOMBAT = the Premier of Randomized Comparison of Bypass Surgery versus Angioplasty Using Sirolimus-Eluting Stent in Patients with Left Main Coronary Artery Disease trial; SYNTAX = the other Synergy between Percutaneous Coronary Intervention with Taxus and Cardiac Surgery trial.


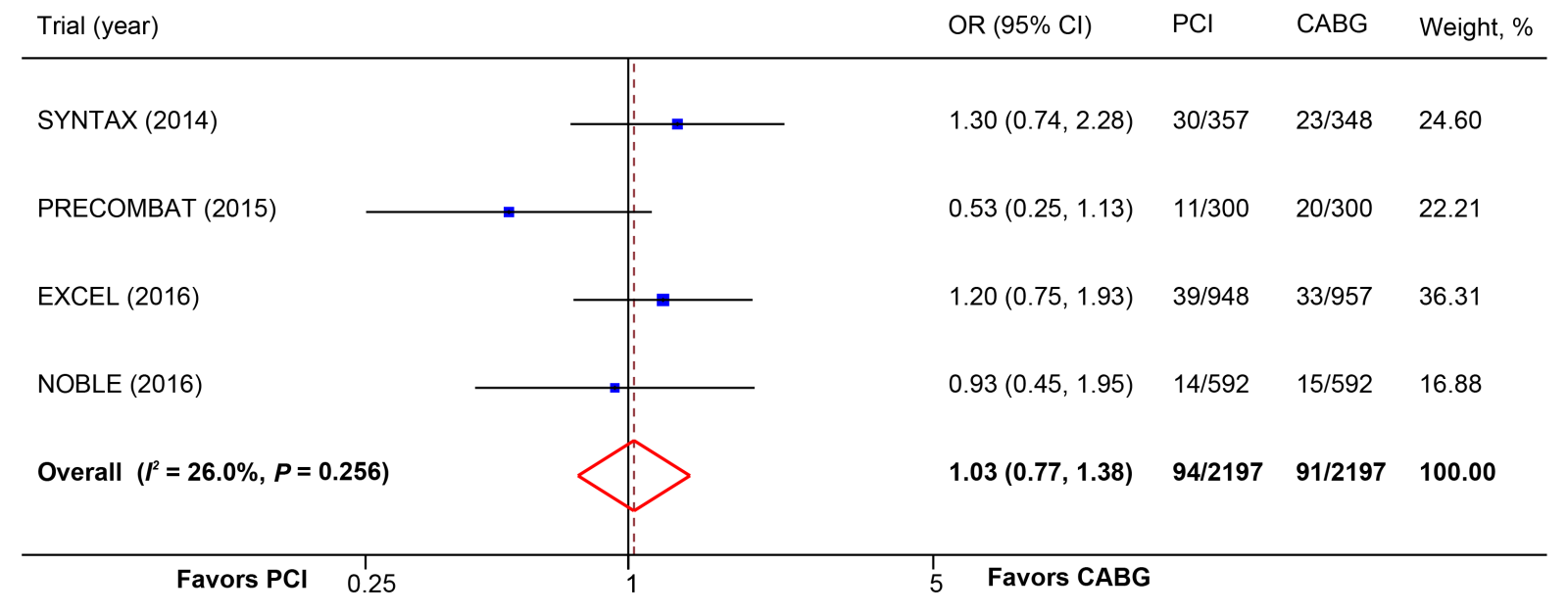


**Figure S3.** Pooled odds ratio for cardiovascular mortality with percutaneous coronary intervention versus coronary artery bypass graft surgery.

CABG = coronary artery bypass graft surgery; CI = confidence interval; OR = odds ratio; PCI = percutaneous coronary intervention. Expanded study abbreviations are as follows: EXCEL = the Evaluation of XIENCE versus Coronary Artery Bypass Surgery for Effectiveness of Left Main Revascularization trial; NOBLE = The Nordic-Baltic-British left main revascularisation study; PRECOMBAT = the Premier of Randomized Comparison of Bypass Surgery versus Angioplasty Using Sirolimus-Eluting Stent in Patients with Left Main Coronary Artery Disease trial; SYNTAX = the other Synergy between Percutaneous Coronary Intervention with Taxus and Cardiac Surgery trial.


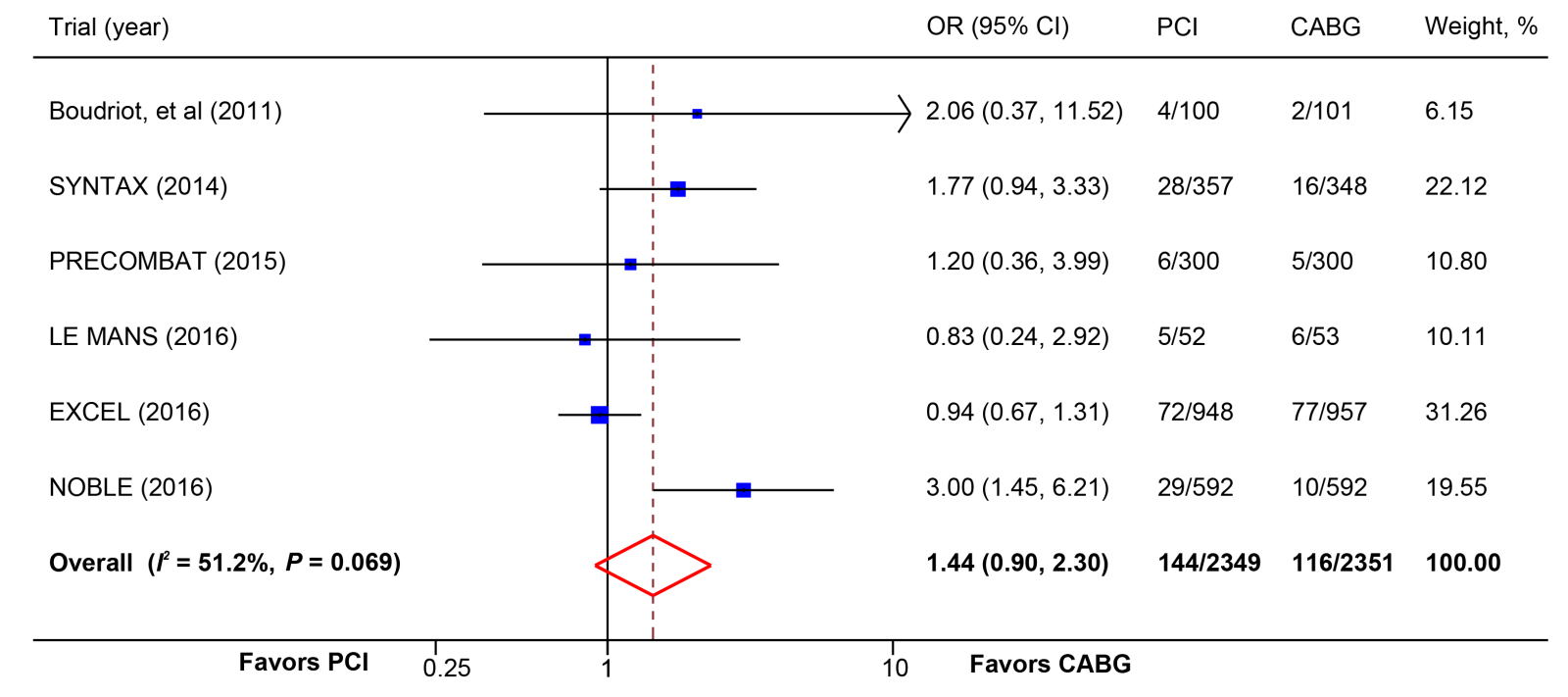


**Figure S4.** Pooled odds ratio for myocardial infarction with percutaneous coronary intervention versus coronary artery bypass graft surgery.

CABG = coronary artery bypass graft surgery; CI = confidence interval; OR = odds ratio; PCI = percutaneous coronary intervention. Expanded study abbreviations are as follows: EXCEL = the Evaluation of XIENCE versus Coronary Artery Bypass Surgery for Effectiveness of Left Main Revascularization trial; LE MANS = Study of UnprotectedLeft Main Stenting Versus Bypass Surgery; NOBLE = The Nordic-Baltic-British left main revascularisation study; PRECOMBAT = the Premier of Randomized Comparison of Bypass Surgery versus Angioplasty Using Sirolimus-Eluting Stent in Patients with Left Main Coronary Artery Disease trial; SYNTAX = the other Synergy between Percutaneous Coronary Intervention with Taxus and Cardiac Surgery trial.


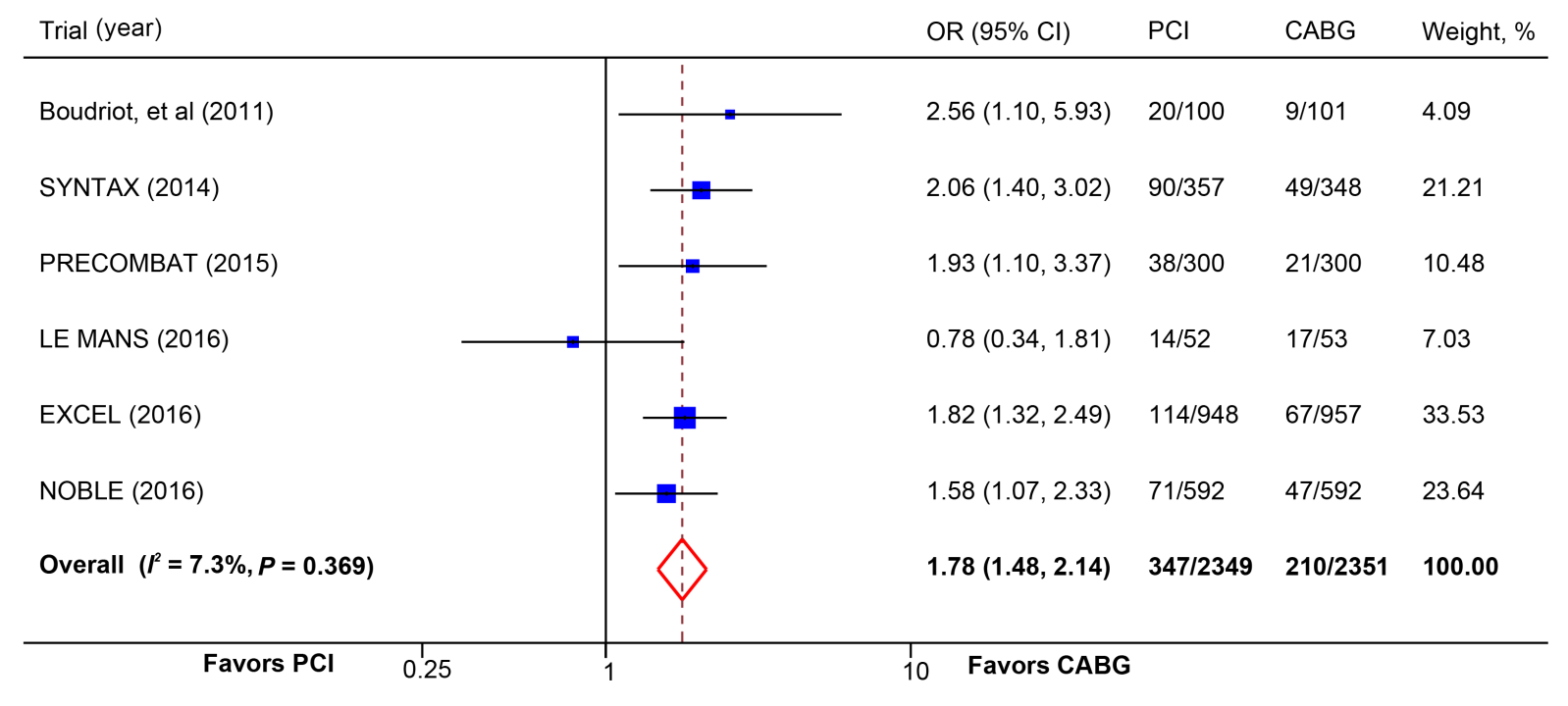


**Figure S5.** Pooled odds ratio for revascularization with percutaneous coronary intervention versus coronary artery bypass graft surgery.

CABG = coronary artery bypass graft surgery; CI = confidence interval; OR = odds ratio; PCI = percutaneous coronary intervention. Expanded study abbreviations are as follows: EXCEL = the Evaluation of XIENCE versus Coronary Artery Bypass Surgery for Effectiveness of Left Main Revascularization trial; LE MANS = Study of UnprotectedLeft Main Stenting Versus Bypass Surgery; NOBLE = The Nordic-Baltic-British left main revascularisation study; PRECOMBAT = the Premier of Randomized Comparison of Bypass Surgery versus Angioplasty Using Sirolimus-Eluting Stent in Patients with Left Main Coronary Artery Disease trial; SYNTAX = the other Synergy between Percutaneous Coronary Intervention with Taxus and Cardiac Surgery trial.


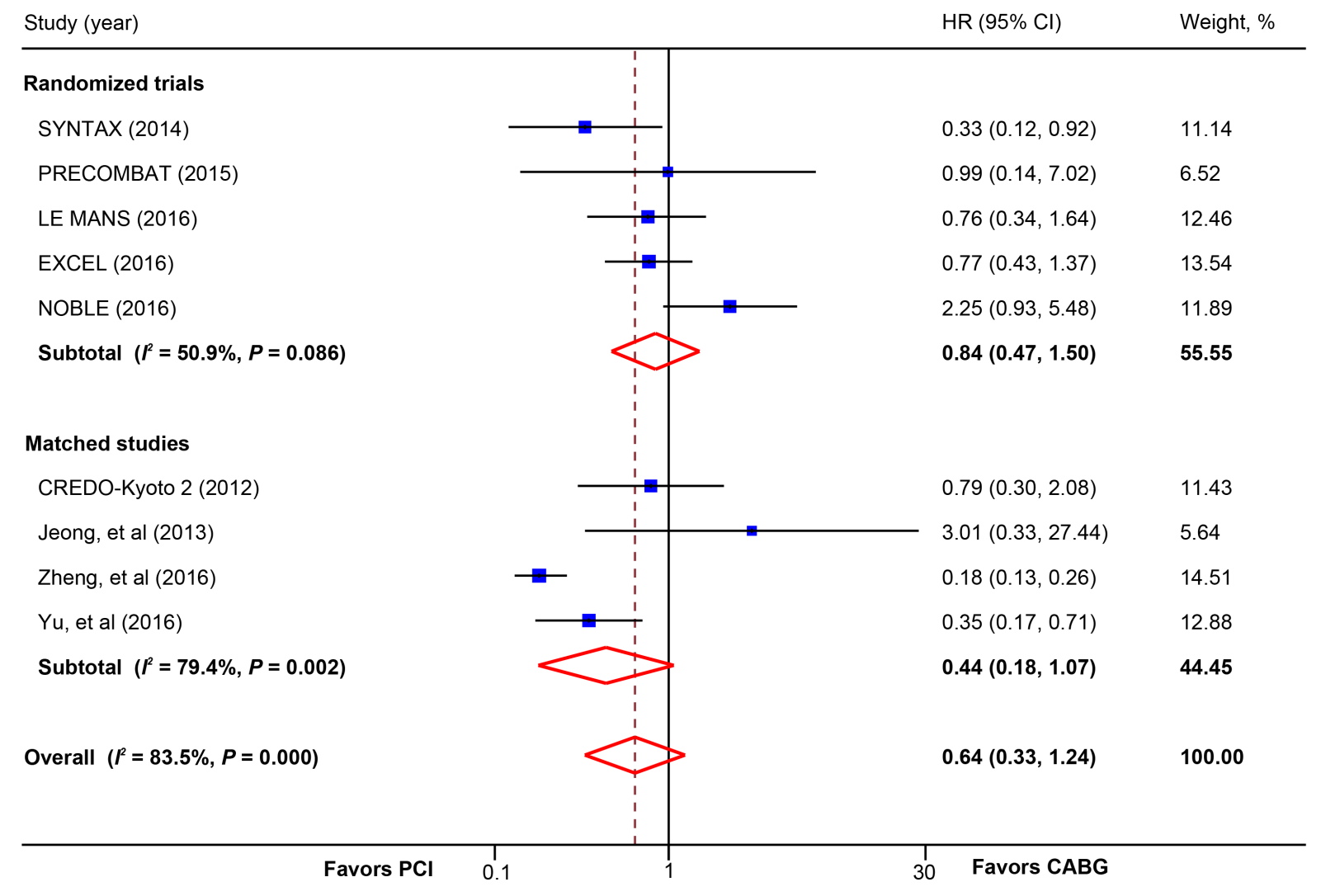


**Figure S6.** Pooled hazard ratio for stroke with percutaneous coronary intervention versus coronary artery bypass graft surgery.

CABG = coronary artery bypass graft surgery; CI = confidence interval; HR = hazard ratio; PCI = percutaneous coronary intervention. Expanded study abbreviations are as follows: CREDO-Kyoto 2 = the Coronary Revascularization Demonstrating Outcome Study in Kyoto (CREDO-Kyoto) PCI/CABG Registry Cohort-2; EXCEL = the Evaluation of XIENCE versus Coronary Artery Bypass Surgery for Effectiveness of Left Main Revascularization trial; LE MANS = Study of UnprotectedLeft Main Stenting Versus Bypass Surgery; NOBLE = The Nordic-Baltic-British left main revascularisation study; PRECOMBAT = the Premier of Randomized Comparison of Bypass Surgery versus Angioplasty Using Sirolimus-Eluting Stent in Patients with Left Main Coronary Artery Disease trial; SYNTAX = the other Synergy between Percutaneous Coronary Intervention with Taxus and Cardiac Surgery trial.


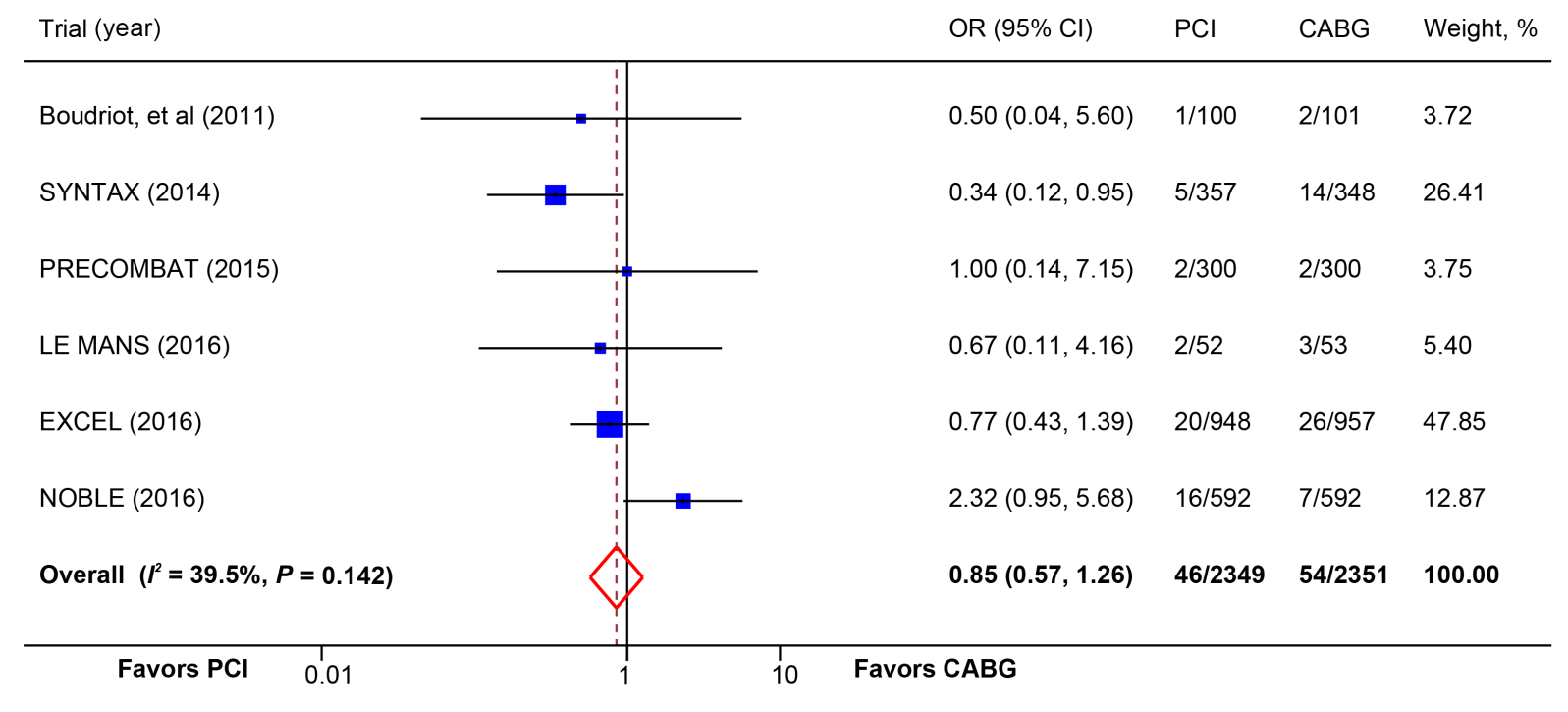


**Figure S7.** Pooled odds ratio for stroke with percutaneous coronary intervention versus coronary artery bypass graft surgery.

CABG = coronary artery bypass graft surgery; CI = confidence interval; OR = odds ratio; PCI = percutaneous coronary intervention. Expanded study abbreviations are as follows: EXCEL = the Evaluation of XIENCE versus Coronary Artery Bypass Surgery for Effectiveness of Left Main Revascularization trial; LE MANS = Study of UnprotectedLeft Main Stenting Versus Bypass Surgery; NOBLE = The Nordic-Baltic-British left main revascularisation study; PRECOMBAT = the Premier of Randomized Comparison of Bypass Surgery versus Angioplasty Using Sirolimus-Eluting Stent in Patients with Left Main Coronary Artery Disease trial; SYNTAX = the other Synergy between Percutaneous Coronary Intervention with Taxus and Cardiac Surgery trial.


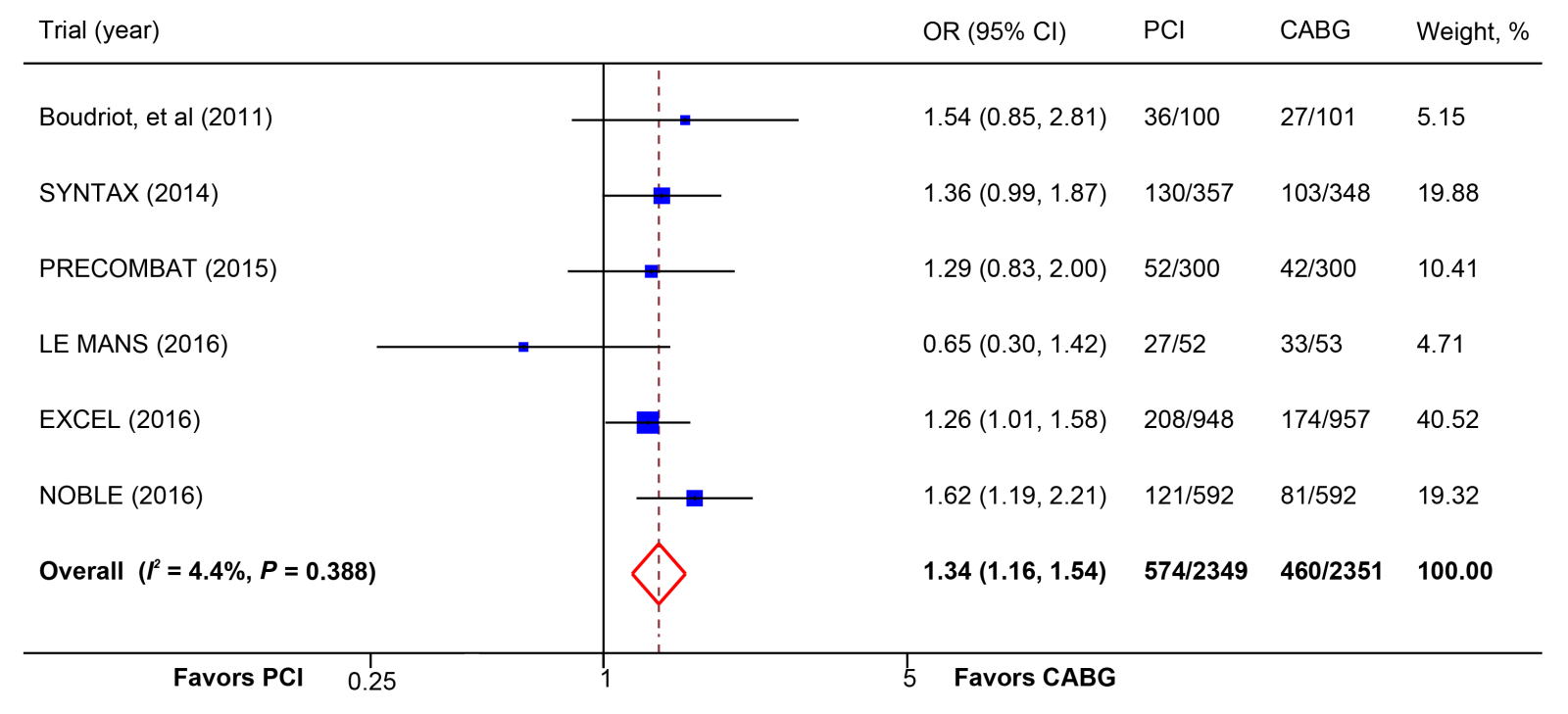


**Figure S8.** Pooled odds ratio for the composite outcome of death, stroke, myocardial infarction and revascularization with percutaneous coronary intervention versus coronary artery bypass graft surgery.

CABG = coronary artery bypass graft surgery; CI = confidence interval; OR = odds ratio; PCI = percutaneous coronary intervention. Expanded study abbreviations are as follows: EXCEL = the Evaluation of XIENCE versus Coronary Artery Bypass Surgery for Effectiveness of Left Main Revascularization trial; LE MANS = Study of UnprotectedLeft Main Stenting Versus Bypass Surgery; NOBLE = The Nordic-Baltic-British left main revascularisation study; PRECOMBAT = the Premier of Randomized Comparison of Bypass Surgery versus Angioplasty Using Sirolimus-Eluting Stent in Patients with Left Main Coronary Artery Disease trial; SYNTAX = the other Synergy between Percutaneous Coronary Intervention with Taxus and Cardiac Surgery trial.


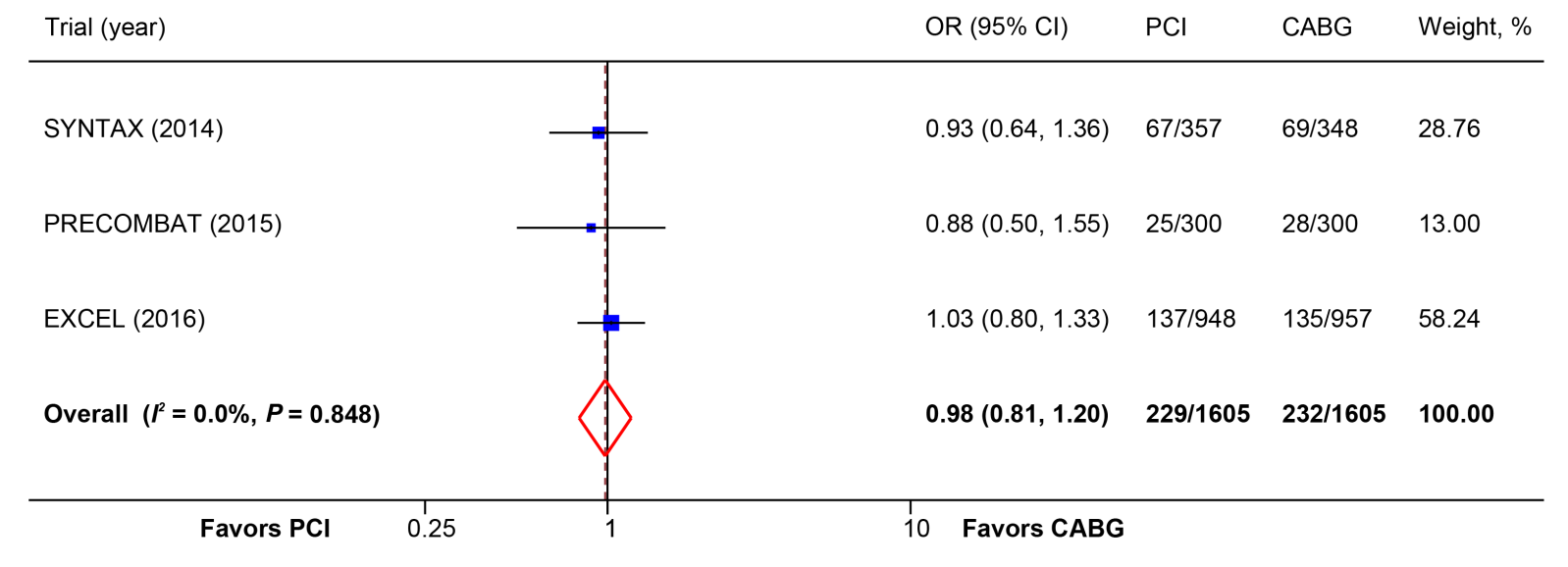


**Figure S9.** Pooled odds ratio for the composite outcome of death, stroke and myocardial infarction with percutaneous coronary intervention versus coronary artery bypass graft surgery.

CABG = coronary artery bypass graft surgery; CI = confidence interval; OR = odds ratio; PCI = percutaneous coronary intervention. Expanded study abbreviations are as follows: EXCEL = the Evaluation of XIENCE versus Coronary Artery Bypass Surgery for Effectiveness of Left Main Revascularization trial; PRECOMBAT = the Premier of Randomized Comparison of Bypass Surgery versus Angioplasty Using Sirolimus-Eluting Stent in Patients with Left Main Coronary Artery Disease trial; SYNTAX = the other Synergy between Percutaneous Coronary Intervention with Taxus and Cardiac Surgery trial.
